# Core–Shell IrPt Nanoalloy on La/Ni–Co_3_O_4_ for High-Performance Bifunctional PEM Electrolysis with Ultralow Noble Metal Loading

**DOI:** 10.1007/s40820-025-01845-7

**Published:** 2025-07-14

**Authors:** Yifei Liu, Xinmeng Er, Xinyao Wang, Hangxing Ren, Wenchao Wang, Feng Cao, Taiyan Zhang, Pan Liu, Yakun Yuan, Fangbo Yu, Yang Ren, Fuqiang Huang, Wenjiang Ding, Lina Chong

**Affiliations:** 1 Center of Hydrogen Science, School of Materials Science and Engineering, Shanghai Jiao Tong University, Shanghai, 2000240 People’s Republic of China; 2 Zhangjiang Institute for Advanced Study (ZIAS), Shanghai Jiao Tong University, Shanghai, 201210 People’s Republic of China; 3 State Key Laboratory for Mechanical Behavior of Materials, School of Materials Science and Engineering, Xi’an Jiao Tong University, Xi’an, 710049 People’s Republic of China; 4 State Key Laboratory of Metal Matrix Composites, Shanghai Jiao Tong University, Shanghai, 200240 People’s Republic of China; 5 Department of Physics, JC STEM Lab of Energy and Materials Physics, City University of Hong Kong, Kowloon Tong, 999077 Hong Kong People’s Republic of China; 6 School of Mechanical Engineering, Shanghai Jiao Tong University, Shanghai, 200240 People’s Republic of China; 7 International Research Center for Renewable Energy, State Key Laboratory of Multiphase Flow in Power Engineering, Xi’an Jiao Tong University, Shaanxi, 710049 People’s Republic of China

**Keywords:** Proton exchange membrane water electrolysis, Bifunctional catalyst, Oxygen evolution reaction, Hydrogen evolution reaction, Core–shell catalyst

## Abstract

The development of highly efficient and durable bifunctional catalysts with minimal precious metal usage is critical for advancing proton exchange membrane water electrolysis (PEMWE). We present an iridium–platinum nanoalloy (IrPt) supported on lanthanum and nickel co-doped cobalt oxide, featuring a core–shell architecture with an amorphous IrPtOx shell and an IrPt core. This catalyst exhibits exceptional bifunctional activity for oxygen and hydrogen evolution reactions in acidic media, achieving 2 A cm^−2^ at 1.72 V in a PEMWE device with ultralow loadings of 0.075 mg_Ir_ cm^−2^ and 0.075 mg_Pt_ cm^−2^ at anode and cathode, respectively. It demonstrates outstanding durability, sustaining water splitting for over 646 h with a degradation rate of only 5 μV h^−1^, outperforming state-of-the-art Ir-based catalysts. In situ X-ray absorption spectroscopy and density functional theory simulations reveal that the optimized charge redistribution between Ir and Pt, along with the IrPt core–IrPtOx shell structure, enhances performance. The Ir–O–Pt active sites enable a bi-nuclear mechanism for oxygen evolution reaction and a Volmer–Tafel mechanism for hydrogen evolution reaction, reducing kinetic barriers. Hierarchical porosity, abundant oxygen vacancies, and a high electrochemical surface area further improve electron and mass transfer. This work offers a cost-effective solution for green hydrogen production and advances the design of high-performance bifunctional catalysts for PEMWE.

**Graphical Abstract:**

**Supplementary Information:**

The online version contains supplementary material available at 10.1007/s40820-025-01845-7.

## Highlights

- Core–shell IrPt nanoalloy on La/Ni–Co₃O₄ achieves unprecedented bifunctional activity (2 A cm^−2^ at 1.72 V) in proton exchange membrane water electrolysis (PEMWE) with ultralow loadings (0.075 mg cm^−2^ Ir/Pt at both electrodes).
- 646-h durability in PEMWE cell (5 μV h^−1^ decay) via IrPt-core@IrPtO_x_-shell synergy, hierarchical pores, and oxygen vacancies for robust electron/mass transfer and active-site stability.
- In situ X-ray absorption spectroscopy combined with density functional theory unveils Ir–O–Pt sites enabling bi-nuclear oxygen evolution reaction and Volmer–Tafel hydrogen evolution reaction mechanisms through optimized Ir/Pt charge redistribution, breaking kinetic limitations.

**Supplementary Information:**

The online version contains supplementary material available at 10.1007/s40820-025-01845-7.

## Graphical abstract

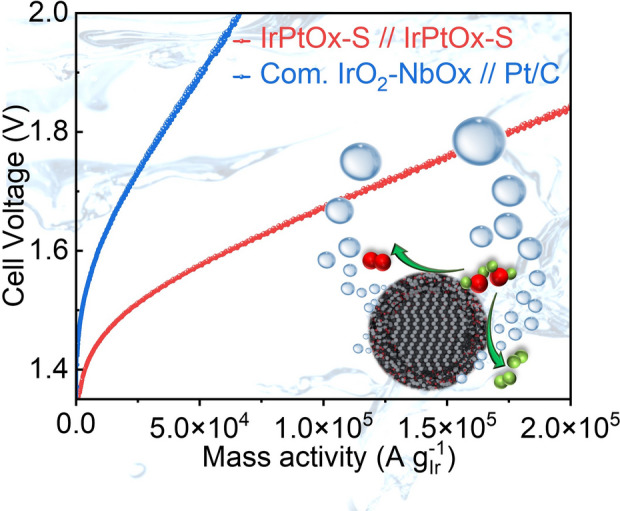

## Introduction

The transition to a sustainable energy future hinges on the development of efficient technologies for green hydrogen production, with proton exchange membrane water electrolysis (PEMWE) emerging as a leading candidate [1]. PEMWE offers a promising pathway to convert and store renewable energy, addressing the global energy crisis and advancing carbon neutrality goals [2–6]. However, the widespread adoption of PEMWE is hindered by the reliance on precious group metals (PGMs), such as platinum (Pt) and iridium (Ir), which are scarce, expensive, and subject to supply chain constraints [7]. Currently, Pt and Ir remain the materials of choice for the hydrogen evolution reaction (HER) and oxygen evolution reaction (OER), respectively, due to their optimal balance of electrocatalytic activity and durability under the harsh acidic and corrosive conditions of PEMWE [8]. Nevertheless, typical Pt loadings at the cathode (~ 0.4 mg cm^−2^) and Ir loadings at the anode (2–4 mg cm^−2^) far exceed the U.S. Department of Energy (DOE) 2026 target of reducing total PGM loading to < 0.5 mg cm^−2^ without compromising performance [9, 10].

The development of bifunctional catalysts capable of catalyzing both OER and HER has gained significant attention, as they simplify electrode manufacturing and reduce costs [11–15]. However, designing such catalysts with significantly reduced PGM loadings while maintaining high activity and stability remains a formidable challenge, particularly under the extreme chemical and electrochemical conditions of PEMWE [16, 17]. While Pt is the most effective HER catalyst, it performs poorly for OER due to the formation of non-conductive PtO₂ under high-voltage conditions, which increases surface resistivity (ρPtO₂ ≈ 10⁶ Ω cm) and reduces activity [18–20]. In contrast, Ir oxides exhibit metallic conductivity, with resistivity approximately 11 orders of magnitude lower than that of Pt oxides, making them more suitable for OER [21]. However, Ir-based catalysts are less effective for HER. To overcome these limitations, researchers have explored Pt–Ir bimetallic systems, which leverage synergistic interactions between Pt and Ir to enhance catalytic activity. For example, Pt nanoparticles dispersed on IrO₂ supports, or IrO₂ formed on Pt black surfaces, have demonstrated excellent bifunctional activity for both OER and HER in three-electrode systems [22–24]. Yim et al. evaluated a series of bifunctional catalysts, including Pt, Pt–Ir, Pt–Ru, and Pt–Ru–Ir, and found that Pt–Ir exhibited the best overall activity, stability, and round-trip efficiency among studies catalysts [25, 26]. Debe et al. developed nanostructured thin films (NSTF) by sputtering Pt–Ir nanoparticles onto organic whisker arrays and evaluated its OER activity which achieved a current density of 2 A cm^−2^ at 1.81 V with a Ir loading of 0.3 mg cm^−2^ at the anode [27]. Despite these advances, the Ir and Pt usage and the longevity of these catalysts are still far from the requirements for the commercialization of PEMWE [10], and they have yet to be tested in a real practical PEMWE cell. Moreover, the use of Pt–Ir catalysts for both the anode and cathode in PEMWE remains underexplored.

The choice of supporting material is critical for achieving high catalytic activity and durability. Supports with high surface area and appropriate porosity can enhance the dispersion of Pt–Ir nanoparticles, improve mass transfer, and mitigate particle agglomeration during operation. For instance, metal oxide-supported Ir oxides exhibit higher activity and durability than carbon-supported counterparts due to strong metal–support interactions (MSI), which tune the electronic configuration of Ir and mitigate over-oxidation and dissolution during OER [28]. Metal–organic frameworks (MOFs) and their derivatives, with their inherently high BET surface area and porosity, demonstrated advantages in mass transfer during OER [29, 30]. Transition metal oxides, such as Co₃O₄ and MoO₃, have shown promise as bifunctional catalysts due to their redox efficiency, tunable electronic properties, and structural flexibility [31–33]. Particularly, Co-based species have demonstrated high activity and long-term durability toward OER in both acidic and alkaline media [34–37]. For example, oxovanadate-doped cobalt carbonate on nickel foam (VCoCO_*x*_@NF) exhibited high overall water splitting performance, including both HER and OER, in an alkaline anion-exchange membrane water electrolyzer (AAEMWE) [34]. Spinel-structured Co₃O₄ demonstrated remarkable stability in acidic environments compared to other cobalt-based materials (e.g., CoSe, CoP, CoB, and CoO) [38]. Doping with elements such as lanthanum (La) and manganese (Mn) can further enhance catalytic activity, durability, and electronic conductivity [39]. Hierarchical porous Co₃O₄ microtube arrays have also demonstrated excellent bifunctional activity and stability for both OER and HER [32]. The affinity between Co and Pt [40] and between Co and Ir [41] suggests that transition metal-doped Co₃O₄ could serve as an effective support for Pt–Ir nanoparticles. Such supports not only provide anchoring sites for nanoparticles but also enable metal/metal oxide support interactions (MMOSI), stabilizing the nanoparticles and enhancing their catalytic performance [28]. Motivated by these findings, we report a bifunctional catalyst design: an iridium–platinum subnanoalloy (IrPt) supported on lanthanum and nickel co-doped cobalt oxide (La–Ni–Co₃O₄). This catalyst features a unique core–shell structure, comprising an amorphous IrPtO_x_ shell and an IrPt core, which synergistically enhances both OER and HER activities while addressing the critical challenges of PGM usage and durability in PEMWE. Unlike our previous studies [29], the introduction of Ni in this work introduces additional defect sites and optimizes the electronic structure of the support, leading to improved performance. Central to the catalyst’s performance is its unique core–shell structure, where the amorphous IrPtO_x_ shell and IrPt core work synergistically to enhance catalytic activity and stability. The amorphous IrPtOx shell provides abundant active sites and facilitates charge transfer, while the IrPt core ensures structural integrity and electronic conductivity. This dual-phase architecture not only optimizes the adsorption energies of reaction intermediates but also mitigates the dissolution and agglomeration of active sites during prolonged operation, addressing a key limitation of traditional PGM-based catalysts.

The Ir–Ir/Pt bond distance in the resulting catalyst (IrPtOx-S, S is the support) (∼2.75 Å) is shorter than that in IrO₂ (∼3.22 Å), enabling a bi-nuclear OER mechanism where two adjacent ∗  = O intermediates directly couple to form O₂ [42]. This pathway bypasses lattice oxygen participation and avoids high-energy intermediates like ∗ OOH, relying solely on ∗ O and ∗ OH as reaction intermediates. By circumventing the scaling relationship limitations of the adsorbate evolution mechanism (AEM) and eliminating metal leaching risks inherent to the lattice oxygen mechanism (LOM) [43], this approach offers enhanced stability. The bi-nuclear mechanism requires precise geometric alignment of active sites (typically 2.4–2.9 Å) [44], a criterion satisfied by the optimized Ir–Pt coordination in our IrPtOₓ-S. For HER in acidic media, the process initiates with the Volmer step (H⁺ + e^−^ → H), forming adsorbed hydrogen (H*) on the catalyst surface. The reaction then proceeds via either the Heyrovsky step (H* + H⁺ + e^−^ → H₂) or the Tafel step (2H* → H₂*). For highly active HER catalysts (e.g., Pt-based systems), the Volmer–Tafel pathway dominates, characterized by Tafel slopes ≤ 29 mV dec^−1^ [45]—a benchmark met by our IrPtO*ₓ*-S catalyst (26 mV dec^−1^). Our IrPtOx-S exhibited exceptional OER and HER activity, with mass activities of 1188.0 ± 30 A g _Ir_^−1^ at 300 mV overpotential for OER and 8725 ± 70 A g _Pt_^−1^ at 100 mV overpotential for HER in 0.1 M HClO₄, representing 33.3-fold and 8.2-fold improvements over commercial IrO₂ and Pt/C benchmarks, respectively. The catalyst also demonstrated remarkable durability, sustaining OER at 10 mA cm^−2^ and HER at − 10 mA cm^−2^ in acidic media for over 1000 h, respectively. When integrated into a membrane electrode assembly (MEA) as both the anode and cathode, the PEMWE cell achieved a current density of 2 A cm^−2^ at 1.72 V with ultralow loadings of 0.075 mg_Ir_ cm^−2^ Ir at the anode and 0.075 mg_Pt_ cm^−2^ Pt at the cathode. To elucidate the underlying mechanisms, we employed density functional theory (DFT) calculations combined with in situ X-ray absorption spectroscopy (XAS) to systematically investigate the catalytic reaction pathways and the origins of the catalyst’s high activity and stability under PEMWE conditions.

## Experimental Section

### Preparation of the Support Material

6 g of Co(NO₃)₂·6H₂O, 1.81 g of La(NO₃)₃·xH₂O, and 2.02 g of Ni(NO_3_)_2_·6H_2_O were dissolved in 150 mL of methanol to form Solution A. Separately, 15 g of 2-methylimidazole was dissolved in 400 mL of methanol to prepare Solution B. Solution A was added dropwise to solution B under continuous stirring at room temperature until a uniform mixture was obtained. The mixture was then sealed and kept at room temperature for 8 h. The resulting La- and Ni-doped Co-MOF was collected by centrifugation, washed three times with methanol, and dried under vacuum overnight. The La-Ni co-doped Co-MOF was carbonized in a tube furnace at 510 °C for 1 h under an argon atmosphere. The carbonized powder was further annealed at 360 °C in air for 4 h to yield the porous support material, La-Ni co-doped Co₃O₄ (denoted as S).

### Preparation of IrPtOx-S, IrOx-S, and Pt–S

38.09 mg of IrCl₃·nH₂O, 43.5 mg of C₁₀H₁₄O₄Pt, 2.7 g of 1,4-phthalaldehyde, and 1.54 g of citric acid were mixed with 64.8 mL of benzyl alcohol and ultrasonicated for 1 h. Subsequently, 140 mg of the support material (S) was added to the mixture and ultrasonicated until a uniform dispersion was achieved. The mixture was then heated at 190 °C for 4 h in an oil bath. After natural cooling, the product was centrifuged, washed three times with a mixture of acetone and ethanol, and freeze-dried in liquid nitrogen. Finally, the material was annealed at 360 °C for 4 h under flowing air to obtain IrPtO_x_-S. The preparation methods for IrOx-S and Pt–S were the same as that for IrPtO_x_-S, except for the absence of C₁₀H₁₄O₄Pt or IrCl₃·nH₂O during the solvothermal reduction step.

### Characterization

The microstructure and morphology of the materials were characterized using spherical aberration-corrected transmission electron microscope (AC-TEM), high-resolution TEM (HRTEM), and high-angle annular dark field-scanning TEM (HAADF-STEM). These analyses were performed on a Talos F200X G2 microscope and a JEOL ARM-200F electron microscope equipped with a spherical aberration corrector (CEOS GmbH), both operated at 200 kV. Energy-dispersive X-ray spectroscopy (EDS) was performed on the same instrument at 200 kV. Powder X-ray diffraction (XRD) patterns were collected using a Mini Flex 600 diffractometer with Cu Kα radiation (*λ* = 1.5406 Å) over a 2*θ* range of 5° to 90°. The surface chemical valence states were analyzed using X-ray photoelectron spectroscopy (XPS) on an AXIS UltraDLD spectrometer with a monochromatic Al Kα source. The binding energies were calibrated using the C 1* s* peak at 284.8 eV to account for charging effects. Raman spectroscopy was performed using a Renishaw inVia Qontor system with a 532 nm laser to analyze chemical bonding. The concentrations of Ir and Pt were determined using inductively coupled plasma mass spectrometry (ICP-MS, Nex ION 2000). The specific surface area and pore size distribution were analyzed using the Brunauer–Emmett–Teller (BET) method via nitrogen adsorption–desorption at 77 K on an Autosorb-iQ3 surface characterization analyzer.

### Ex-Situ and In-Situ X-Ray Absorption Measurements

X-ray absorption spectra (XAS) were collected at two beamlines: BL14W1 at the Shanghai Synchrotron Radiation Facility (SSRF, China) using a Si (111) double-crystal monochromator, and the Optique Dispersive EXAFS (ODE) line station at the SOLEIL Synchrotron (France). The energy was set to 11,215 eV for the Ir L_III_ edge, 11,564 eV for the Pt L_III_ edge, and 7709 eV for the Co K edge. Spectra were collected at room temperature in fluorescence mode at BL14W1 and in transmission mode at ODE. Reference materials, including commercial IrO₂, Ir black, IrO₂/NbOx, Pt/C, PtO₂·xH₂O, Co₃O₄, and CoO, were measured for comparison. XAS data were processed and analyzed using Athena and Artemis software following standard procedures. Wavelet transform–extended X-ray absorption fine structure (WT-EXAFS) for k^2^-weighted EXAFS oscillations enables the identification of bond pair contributions to the Fourier transform peaks. For in situ XAS measurements, a custom-designed electrochemical cell was used. The working electrode (WE) consisted of a catalyst-modified graphene sheet, while a Pt wire and Hg/Hg₂SO₄ served as the counter electrode (CE) and reference electrode (RE), respectively. The electrolytes were O₂-saturated 0.1 M HClO₄ for OER and H₂-saturated 0.5 M H₂SO₄ for HER. Additionally, X-ray absorption near-edge structure (XANES) spectra at the O K-edge were collected in total electron yield (TEY) mode using soft X-ray absorption spectroscopy at the BL08U1-A beamline of SSRF, with the absorption energy set at 543.1 eV.

### Electrochemical Characterization

The electrochemical performance of the catalysts was evaluated using a standard three-electrode system with an electrochemical workstation (WaveDriver200, Pine, PHYCHEMI). For OER measurements, the electrolyte was O_2_-saturated 0.1 M HClO_4_, the WE was a catalyst-coated gold disk electrode (GDE, 5.0 mm diameter, Pine, PHYCHEMI), the CE was a gold wire, and the RE was Hg/Hg₂SO₄ (Pine Instruments).The catalyst ink was prepared by ultrasonically dispersing 2 mg of catalyst in a mixture of 0.4 mL deionized water, 0.3 mL isopropanol, and 3 μL 5% Nafion. 10 µL of the ink was drop-cast onto the gold disk, with an Ir loading of 10 µg cm^−2^. For the support material (S), the loading was 150 µg cm^−2^, and for commercial IrO₂, the Ir loading was 44 µg cm^−2^.

For HER measurements, the electrolyte was H_2_-saturated 0.1 M HClO_4_ and 0.5 M H_2_SO_4_ solution, respectively. WE was a catalyst-coated glassy carbon disk electrode (GDE, 5.0 mm diameter, Pine Instruments), the CE was a graphite electrode, and RE was Hg/Hg_2_SO_4_ (Pine Instruments) The catalyst ink was prepared by ultrasonically dispersing 2 mg of catalyst and 0.2 mg of carbon in a mixture of 0.4 mL deionized water, 0.3 mL IPA, and 0.003 mL 5% Nafion. 10 µL of the ink was drop-cast onto the glassy carbon disk, with a Pt loading of 0.010 mg cm^−2^. For commercial Pt/C, the Pt loading was also 0.020 mg cm^−2^. All potentials were referenced to the reversible hydrogen electrode (RHE).

For OER testing, linear sweep voltammetry (LSV) was performed in O₂-saturated 0.1 M HClO₄ from 1.0 to 2.0 V (vs. RHE) at a scan rate of 2 mV s^−1^ with 85% iR compensation. For HER testing, LSV was performed in H₂-saturated 0.1 M HClO₄ from 0 to − 0.5 V (vs. RHE) at a scan rate of 2 mV s^−1^ with 85% iR compensation. Before LSV, the catalysts were stabilized by multiple cyclic voltammetry (CV) cycles at 100 mV s^−1^. Each sample was tested at least three times for reproducibility.

OER overpotential (η_OER_) was calculated at 10 mA cm^−2^ using η_OER_ = E_RHE_–1.23 V. HER overpotential (η_HER_) was calculated at − 10 mA cm^−2^ using η_HER_ = E_RHE_–0 V. The Tafel slope was determined from the Tafel equation, η = b log j + a, where b is the Tafel slope and j is the current density. The electrochemical surface areas (ECSAs) of the samples in units of m^2^ g^−1^ were calculated using the equation: ECSA = Q/(г.L), where Q is the integrated charge density (C cm^−2^), Γ is the specific charge required to oxidize/reduce a monolayer of the adsorbed species (210 μC cm^−2^ for Pt, 596 μC cm^−2^ for IrO₂) [46], and L is the Pt or Ir loading of the electrode (g m^−2^). Hydrogen underpotential deposition (H-UPD) method was used for IrPtO_x_-S, Pt–S, and Com. Pt/C. Redox-active surface charge method was used for IrO_x_-S and Com. IrO_2_. The turnover frequency (TOF) was calculated based on equation: TOF = *j* × *A*/(4 × *F* × *n*), where *j* is the current density, *A* is the electrode area, *F* is the Faraday constant (96,485 C mol^−1^), and n is the number of Ir or Pt atoms participating in the reaction (obtained from ECSA). The accelerated stress test (AST) for OER was performed in O₂-saturated 0.1 M HClO₄ from 1.3 to 1.8 V (vs. RHE) at 100 mV s^−1^ for 40,000 cycles. The AST for HER was performed in H₂-saturated 0.5 M H₂SO₄ from 0 to − 0.8 V (vs. RHE) at 100 mV s^−1^ for 40,000 cycles. LSV polarization curves were recorded before and after AST. Chronoamperometric measurements were conducted at constant current density of ± 10 mA cm^−2^ for OER and HER, respectively. The electrolytes were refreshed periodically.

### PEMWE Performance Measurements

PEMWE cell performance was evaluated using a 600 ETS instrument (Scribner). Membrane electrode assemblies (MEAs) were prepared using the catalyst-coated membrane (CCM) method with GORE membranes (GORE-SELECT® M275.80, W. L. Gore & Associates, proton resistance of 57 mΩ cm^2^ at 80 °C and 100% relative humidity), Ti felt (STi025-Pt0.5) as anodic gas diffusion layer (GDL), and carbon paper (Toray, TGP-H-060) as cathodic GDL. For reproducibility, at least three MEAs were assembled and tested for each sample. For MEA fabrication, the ink was prepared by ultrasonically dispersing the catalyst, 5 wt% Nafion ionomer, IPA, and deionized water, followed by spraying onto the membrane. The active geometric area of the MEA is 5 cm^2^. For MEAs with IrPtOx-S as the anode, cathode, or both, Ir loadings were set at 0.075 mg cm^−2^ at anode, and Pt loadings were set at 0.075 mg cm^−2^ at cathode, respectively. For MEAs with IrO_2_/NbO_2_ (anode, Umicro) and Pt/C (cathode, TKK), the Ir loadings were set at 0.2 mg cm^−2^, and Pt loadings were set at 0.3 mg cm^−2^, respectively. Polarization curves were recorded at a scan rate of 0.008 A min^−1^ from 0 to 20 A at 80 °C. The long-term durability measurements of MEAs were conducted at constant current density of 1.8 A cm^−2^.

### ICP-MS Measurements and Lifetime Estimation

The composition of the catalyst and the dissolved metal ions were analyzed using an inductively coupled plasma mass spectrometer (ICP-MS, Agilent 7900). Prior to measurements, the ICP-MS instrument was calibrated by using multi-element solutions containing Ir, Pt, Co, and La with concentrations of 0.2, 1, 5, 20, and 200 ppb, respectively. To measure the dissolved metals during durability tests at a constant current density of 10 mA cm^−2^, the electrolytes were replaced after each collection for subsequent ICP-MS analysis. At every interval, a portion of the electrolyte was collected, and the leached metal elements were quantitatively measured. The average dissolution rate of the leached elements was calculated using the following equation:

1 $$v = \frac{{V \cdot C_{ICP } }}{{t \cdot m \cdot {\text{wt}}\% }}$$

where *V* is the volume of the electrolyte, *C*_ICP_ is the concentration of elements leached into the electrolyte, *t* is the time interval between two electrolyte collections, m is the total weight of the electrode, and wt% is the weight percentage of the leached element in the catalyst.

The stability number (S1) was determined by the following equation [47]:

2 $$S1 = \frac{{n_{{{\text{Oxygen}}\left( {{\text{OER}}} \right)}} }}{{n_{{{\text{Ir}}\left( {{\text{dissolved}}} \right)}} }}$$

The catalyst lifetime was estimated using the equation [47]:

3 $$t_{{{\text{lifetime}}}} = \frac{S \cdot Z \cdot F \cdot m}{{j \cdot M}}$$

where $t_{lifetime}$ is the lifetime of the catalyst, *S* is the stability number, *Z* is the number of electrons per evolved O_2_, *F* is the faraday constant, m is the loaded mass of Ir or Pt (g cm^−2^), *j* is the applied current density (A cm^−2^), and *M* is the molar mass of Ir or Pt.

### ^18^O MS Test

A working electrode was prepared by drop-casting the catalyst ink onto a glassy carbon electrode (GDE). The ink was prepared by dispersing 6 mg of catalyst powder in a mixture of 31 µL Nafion and 2 mL isopropanol, followed by ultrasonication for 30 min. Electrochemical measurements were performed using an Autolab electrochemical workstation in a three-electrode cell, consisting of the catalyst-modified working electrode, an Hg/Hg₂SO₄ reference electrode, and a Pt wire counter electrode. To label the catalyst with ^1^⁸O, the working electrode was first operated at a constant current density of 15 mA cm^−2^ for 10 min in 0.1 M HClO₄ electrolyte prepared with H₂^1^⁸O (Sinopharm Chemical Reagent Co., Ltd). The electrode and cell were then rinsed with deionized water to remove any residual H₂^1^⁸O. Subsequently, the electrode was operated at 15 mA cm^−2^ for an additional 30 min, during which mass-selected product signals (*m*/*z* = 32 and 34) were collected every 5 min using a GC–MS system with an ionization voltage of 70 eV. Each data point represents the average of five measurements, and all results were background-subtracted.

### DFT Calculations

Periodic density functional theory (DFT) calculations were performed using the projected augmented wave (PAW) method implemented in the Vienna Ab-initio Simulation Package (VASP) [48]. The calculations employed the Perdew–Burke–Ernzerhof (PBE) exchange–correlation functional within the generalized gradient approximation (GGA), supplemented with van der Waals (vdW) corrections [49, 50]. A set of 3 × 6 × 1 and 6 × 9 × 1 Monkhorst–Pack grids were used for geometric optimization, with a cutoff energy of 400 eV for plane-wave basis setup. The maximum atomic forces and energy were ≤ 0.01 eV Å^−1^ and 10^–5^ eV cell^−1^, respectively. For the slab model, a 15 Å vacuum layer was added along the *z*-axis to prevent interactions between periodic images. To account for the strong on-site Coulomb repulsion among transition metals, the GGA + U approach was employed by introducing a Hubbard-like repulsion term (Ueff = U–J) [51]. The Ueff value for Co was set to 3.3 eV.

The reaction mechanism was evaluated using a three-state diagram, including an initial state (H⁺), an intermediate state (H*), and the final product (½ H₂). For the oxygen evolution reaction (OER), the intermediates included OH*, O*, and OOH*. Based on in situ XANES results, projected density of states (pDOS) analysis, and TEM images, an Ir–O–Pt model was used to simulate the active sites for OER and HER.

The free energy of adsorption (Δ*G*_adsorbate_*) was used as a descriptor to evaluate catalytic activity. The free energies of intermediates at 298 K were calculated using the equation:

4 $$\Delta G_{{{\text{adsorbate}}*}} = \Delta E_{{{\text{adsorbate}}*}} + \Delta E_{{{\text{ZPE}}}} - T\Delta S_{{\text{H}}} + eU$$

where *ΔE*_adsorbate*_ is the binding energy of the absorbed intermediates, *ΔE*_ZPE_ is the zero-point energy changes, *TΔS*_H_ is the entropy at room temperature (*T* = 298.15 K), and eU is the applied potential. The limiting potential (*U*_L_) and overpotential (*η*) were determined using:

5 $$U_{L} = \Delta G_{{{\text{max}}}} /{\text{ne}}$$

6 $$\eta = U_{{{\text{ideal}}}} - U_{{\text{L}}}$$

where Δ*G*_max_ is the free energy change of the rate-determining step (RDS), *n* and *e* are the number of electrons and electron charge [52], respectively. *U*_ideal_ is the theoretical potential from a thermodynamic perspective.

## Results and Discussion

### Synthesis and Characterization of IrPtO_x_-S

**Fig. 1:**
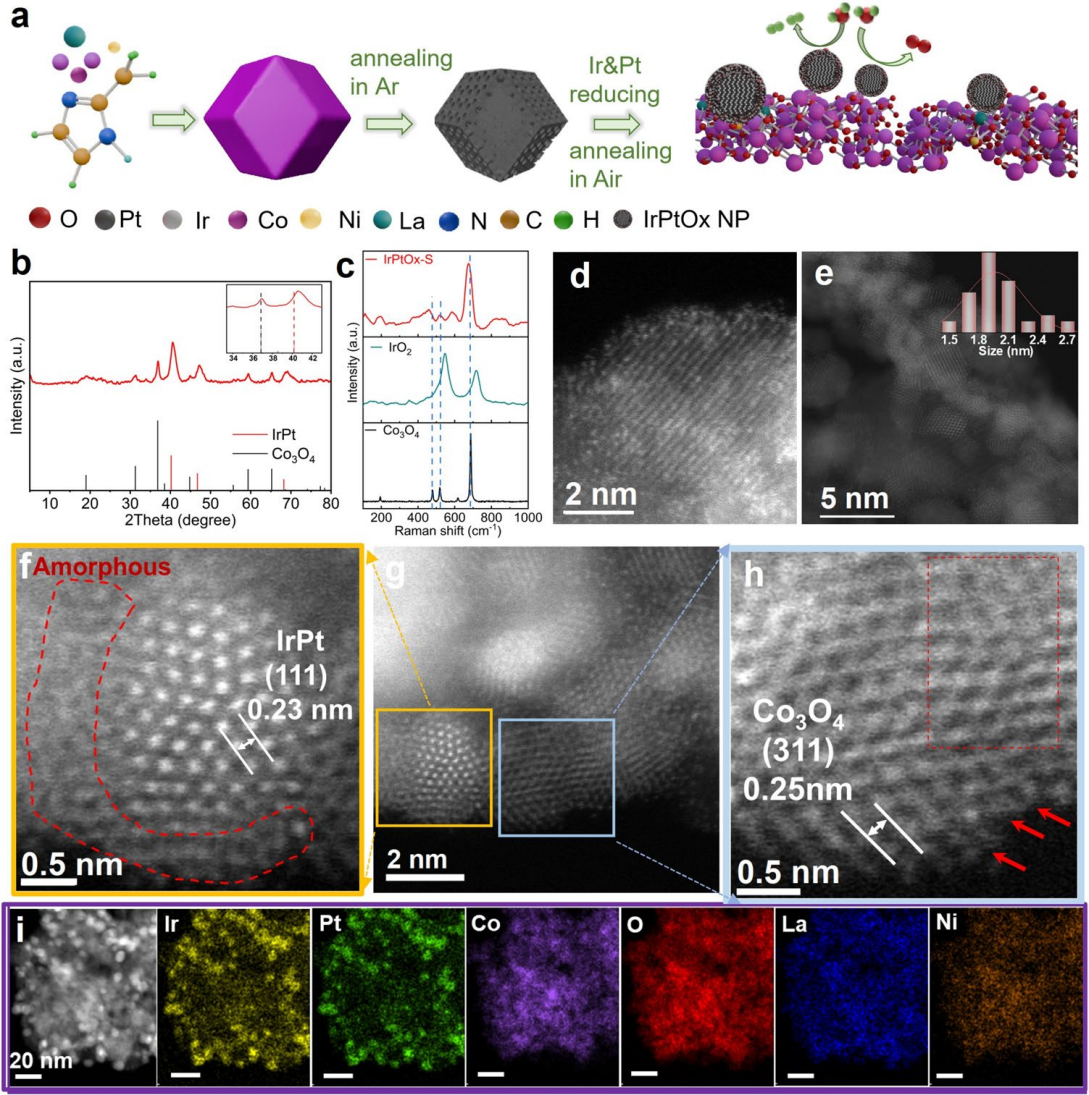
Structure characterizations of IrPtOx-S:** a** illustration of IrPtOx-S synthesis. **b** XRD pattern. **c** Raman spectrum. IrO_2_ and Co_3_O_4_ were the references. **d** AC-TEM image of support material (S). **e** Particle size distribution of IrPt. **f**, **h** HRTEM images of individual particles indicated in g). **g** HRTEM image of IrPtOx-S. **i** HAADF-STEM and corresponding elemental mapping images of Ir, Pt, Co, La, Ni, and O

IrPtOx-S was synthesized through a solvothermal method, involving the in situ reduction of Pt and Ir on the surface of a carbonized La- and Ni-doped cobalt metal–organic framework, followed by calcination in flowing air at 360 °C. During the calcination in flowing air, the carbon template was completely removed, leaving a highly porous and conductive oxide support. A schematic of the synthesis process is shown in Fig. 1a.

XRD analysis confirmed the formation of an Ir–Pt alloy and spinel-structured Co₃O₄ (Fig. 1b). The peaks corresponding Ir–Pt were shifted to higher angles (Fig. 1b, inset), indicating compressive strain in the Ir–Pt particles. Such lattice strain is known to enhance catalytic activity by optimizing the adsorption energy of intermediates [53]. Raman spectroscopy revealed lattice expansion induced by transition metal doping, as evidenced by red-shifted Raman bands and broadened peaks compared to pure Co₃O₄ (Fig. 1c). A broad peak in the 549–625 cm^−1^ range was attributed to amorphous IrOx [54], suggesting the surface of Pt–Ir alloy may be covered by amorphous Ir-O phases. These structural modifications are critical for improving the catalyst’s electronic properties and reactivity.

The catalyst was highly porous, as indicated by scanning electron microscopy (SEM) (Fig. S1). TEM images (Fig. 1d) clearly distinguished individual transition metal dopants, e.g., La, Ir, and Pt uniformly dispersed within the Co₃O₄ lattice, as heavier atoms appeared brighter due to their higher atomic numbers [55]. The average particle sizes of IrPt alloy and the whole catalyst, determined by measuring hundreds of particles across different regions, were estimated to be 2.1 and 3.0 nm, respectively (Figs. 1e and S2), highlighting the nanoscale nature of the catalyst. HR-TEM images revealed the surface of IrPt alloy being covered by an amorphous species with thickness of ~ 2 nm, which can be ascribed to the amorphous IrOx/IrPtO_x_ based on Raman result (Fig. 1f). Amorphous IrO_x_ is known to possess abundant metal dangling bonds and flexible unsaturated electronic configurations, which enhance orbital coupling with intermediates, facilitate charge transfer, and improve catalytic activity [56]. The lattice spacings of 2.3 and 2.5 Å were assigned to the Ir–Pt (111) and Co₃O₄ (311) planes, respectively (Figs. 1f, h and S3). The Ir–Pt (111) spacing was smaller than that of pure Ir–Pt (111) (2.4 Å) [57], suggesting ~ 4% lattice compression, consistent with the XRD results. Conversely, the Co₃O₄ (311) spacing was expanded compared to pure Co₃O₄ (311) (2.4 Å) [58], indicating successful doping of transition metals into the Co₃O₄ lattice. These lattice strain and defects induced by doping optimize the adsorption energy of active sites for intermediates, enhancing catalytic activity [53]. Additionally, the size and oxidation state differences among different dopants create lattice distortions, which mitigate element dissolution during corrosive catalytic processes [59]. Lattice expansion and disordered edges, induced by interactions between components of different sizes, were clearly visualized (Fig. 1h). These features modify the d-band and electronic structures of active sites, improving catalytic activity and mass transfer [56]. Elemental mapping confirmed the uniform distribution of Co, O, La, Ni, Ir, and Pt throughout the catalyst (Figs. 1I and S4–S5) and supported the formation of Ir–Pt alloy nanoparticles (Fig. 1I, Ir and Pt images). STEM-EDS line-scan profiles further confirmed the formation of IrPt alloy core with particle size being around 2–3 nm and IrPtOx shell with thickness of 2–3 nm (Fig. S4b). The concentrations of Ir and Pt were quantified to be 6.738 and 6.779 wt% by ICP-MS (Table S1). N₂ adsorption–desorption analysis revealed a BET surface area of 144.5 m^2^ g^−1^ and an average pore diameter of 5.5 nm (Fig. S6, Table S2). The hierarchical porosity and high surface area of the catalyst facilitate mass transport and expose abundant active sites, further enhancing its performance.

**Fig. 2:**
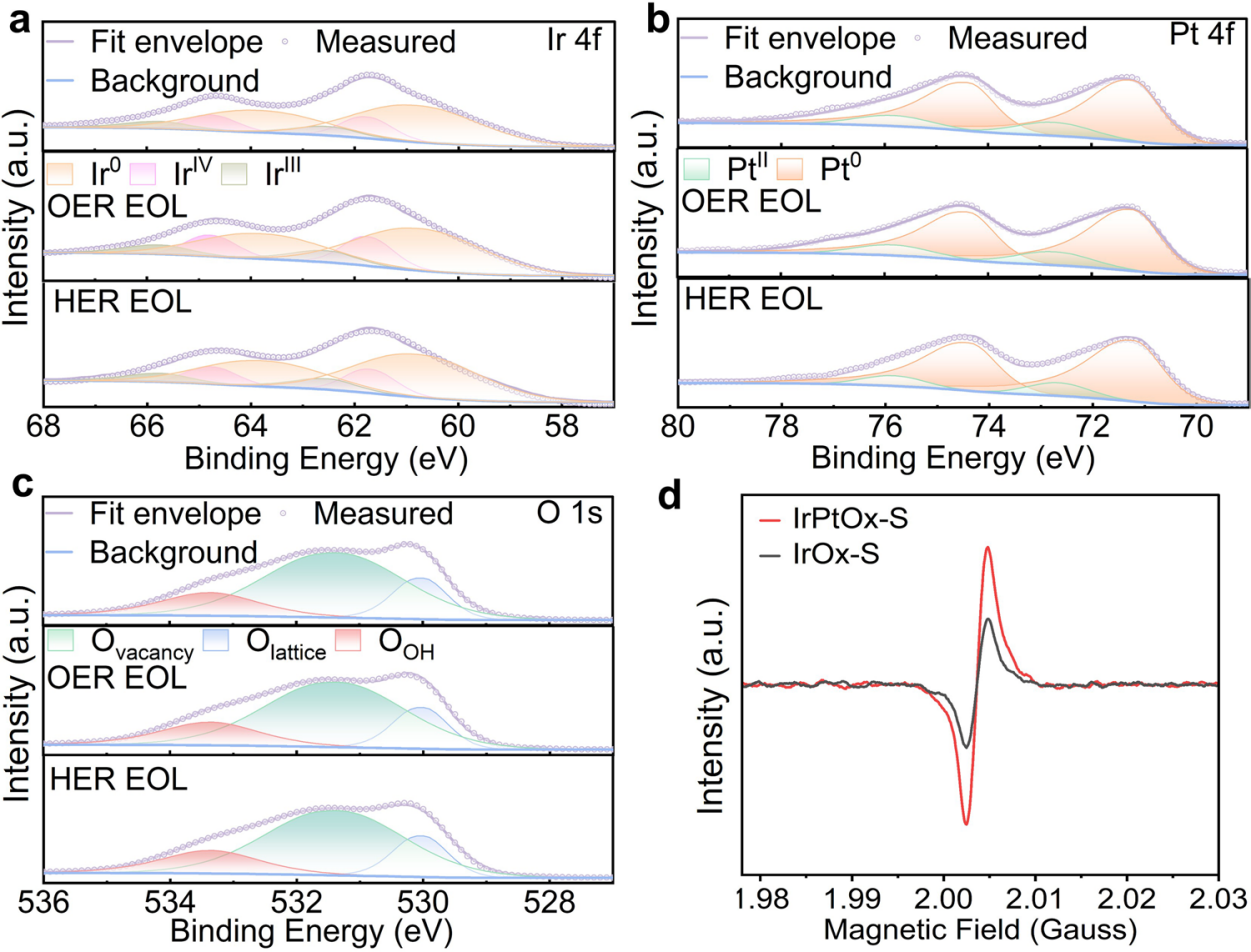
Chemical and structural analysis of IrPtOx-S. XPS spectra of **a** Ir 4*f*, **b** Pt 4*f*, **c** O 1*s* of fresh sample (top), and the samples peeled off from the MEA at the end of life (EOL) test of OER (anode) and HER (cathode), respectively. **d** EPR spectra of IrPtOx-S and IrOx-S

XPS was employed to investigate the surface structure and oxidation states of each element in the catalyst. The survey spectrum confirmed the presence of Co, La, Ni, Pt, Ir, and O (Fig. S7a). The Ir 4*f* spectrum was deconvoluted into three sets of doublets: 60.8/63.8 eV (Ir⁰), 61.8/63.6 eV (Ir^4^⁺), and 62.4/65.3 eV (Ir^3^⁺) (Fig. 2a) with the Ir⁰ as the dominate [60]. The presence of Ir⁰ supports the formation of a Pt–Ir alloy, while the presence of Ir^3^⁺ and Ir^4^⁺ suggests the coexistence of amorphous Ir-O phase on the metal surface, in line with XRD and Raman results. The Pt 4*f* spectrum featured four peaks: 71.4 eV (Pt^0^ 4*f*₇/₂), 74.7 eV (Pt⁰ 4*f*₅/₂), 72.16 eV (Pt^2^⁺ 4*f*₇/₂), and 75.78 eV (Pt^2^⁺ 4*f*₅/₂) with Pt⁰ as the dominate [61] (Fig. 2b). XPS results further suggest that the surface of the IrPt alloy is covered by Ir–Pt–O phase. Compared to IrO_x_-S and Com. IrO_2_, the binding energy of Ir 4*f* in IrPtO_x_-S indicates that the introduction of Pt mitigates Ir oxidation during annealing, favoring Pt–Ir alloy formation (Fig. S8a). The Pt⁰ 4*f* binding energy in IrPtO_x_-S was positively shifted by 0.3 eV relative to pure Pt⁰, indicating electron redistribution between Pt and Ir due to their differing electronegativities (Fig. S8b).

The O 1*s* spectrum was deconvoluted into three peaks: 530.1 eV (lattice oxygen), 531.4 eV (oxygen vacancies, Vₒ), and 533.3 eV (surface hydroxyl groups) [62] (Fig. 2c). The high concentration of oxygen vacancies was further confirmed by electron paramagnetic resonance (EPR) spectroscopy, which showed a stronger signal at g = 2.003 compared to that of catalyst without Pt (IrO_x_-S) [63, 64] (Fig. 2d). The introduction of Pt significantly increased the oxygen vacancy concentration, driven by charge compensation effects as lower-valent Pt substitutes for higher-valent Ir/Co. This substitution necessitates the removal of oxygen atoms to maintain charge neutrality, creating a highly active and conductive catalyst surface. These observations strongly support the idea that the coexistence of multiple elements (La, Ni, Co, Pt, and Ir) induces a redistribution of electronic coupling among the metal ions, enhancing the material’s catalytic properties. Additionally, oxygen vacancies enhance electron conductivity and catalytic activity by facilitating electron transfer [65]. The Co 2*p* spectrum exhibited characteristic peaks of spinel-structured Co₃O₄, with Co^3^⁺ 2*p*₃_/_₂ at 779.8 eV, Co^2^⁺ 2*p*₃_/_₂ at 781.0 eV, and a Co^2^⁺ satellite peak at 785.8 eV [66] (Fig. S7b). The Co^3^⁺/Co^2^⁺ ratio in the catalyst was lower than that of pure Co₃O₄, suggesting partial substitution of Co^3^⁺ by dopants such as Ir^3^⁺, La^3^⁺, or Ni^3^⁺. XPS spectra also revealed that La was partially attached to hydroxyl groups (Fig. S7c). It is well-known that hydroxyl groups on the surface of nanomaterials can enhance pseudo-capacitance, thereby exposing more accessible active sites and boosting catalytic activity [47].

**Fig. 3:**
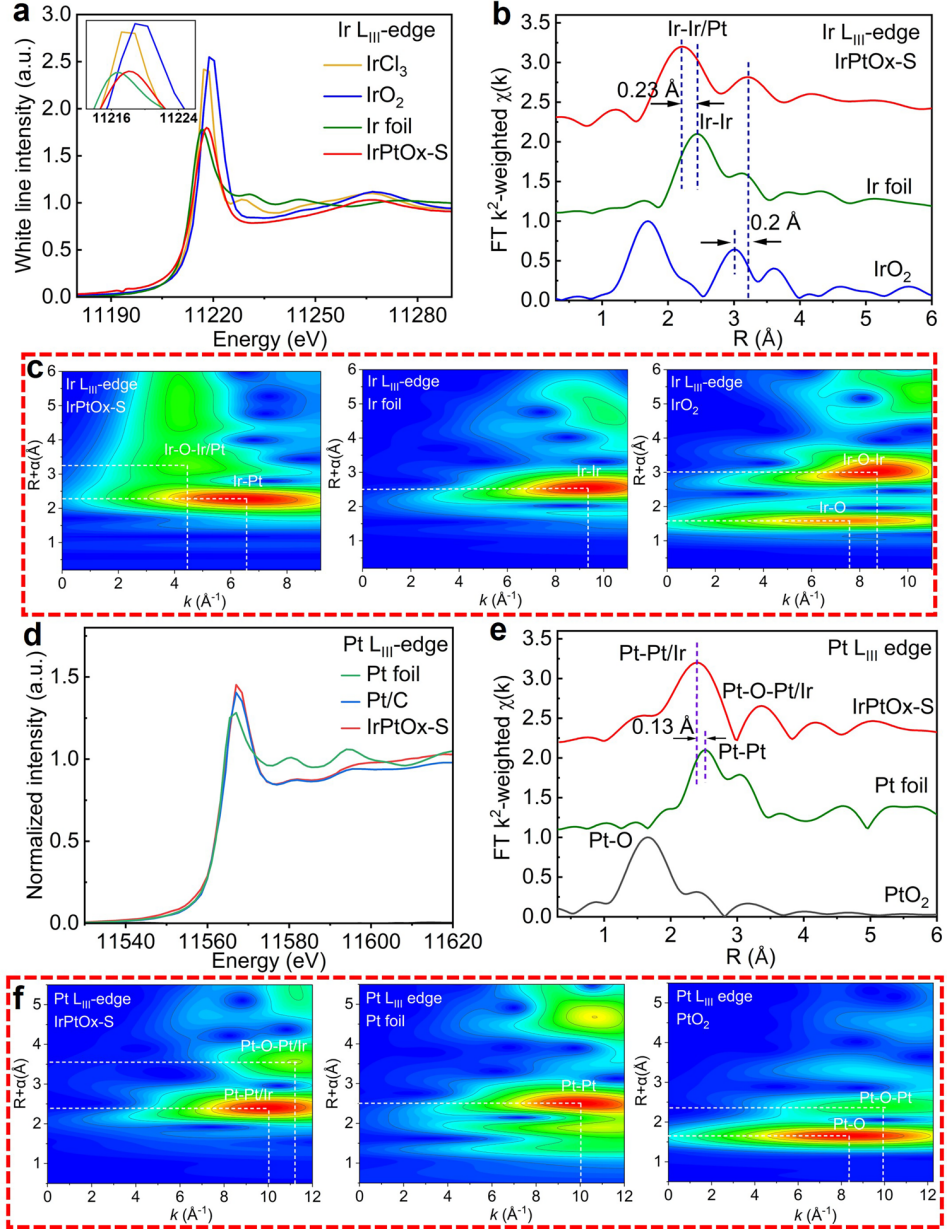
Electronic and atomic structure analysis of IrPtOx-S. **a** XANES spectra, **b** k^2^-weighted FT EXAFS spectra, and **c** wavelet transform images of Ir L_III_-edge EXAFS. IrCl_3_, IrO_2_, and Ir foil were the references. **d** XANES spectra, **e** k^2^-weighted FT EXAFS spectra, and **f** wavelet transform images of Pt L_III_-edge EXAFS. Pt foil, Pt/C, and PtO_2_ were the references

XAS spectra including XANES and EXAFS were performed at Ir L_III_, Pt L_III_, and Co K edges to investigate the electronic structure and local atomic environment of IrPtOx-S. The XANES spectrum at Ir L_III_ edge reflects the unoccupied 5*d* states, where the peak position shifts to higher energies and the integrated intensity of the white line increases with the number of *d* holes (*d* vacancies) [60]. Figure 3a shows the XANES spectrum at the Ir L_III_ edge for IrPtO_x_-S, along with references for IrCl₃, IrO₂, and Ir foil. The absorption energy of Ir L_III_ edge in IrPtO_x_-S is close to that of Ir foil, but the white line peak is shifted by 1.5 eV to higher energy, positioned between IrCl₃ and IrO₂ (inset of Fig. 3a). This indicates that Ir in IrPtO_x_-S exists in a mixed oxidation state ranging from 0 to + 4.

The EXAFS spectrum at the Ir L_III_ edge shows two distinct peaks in R space (Fig. 3b). The first peak at 2.30 is 0.23 Å shorter than the Ir–Ir bond in Ir foil (2.53 Å), suggesting the formation of an Ir–Pt bond and IrPt alloy. The shorter Ir–Pt bond distance may be attributed to the compression of the Ir–Pt bond, as shown in XRD pattern. The second peak at 3.21 Å is assigned to Ir–O–Pt, distinguishable from the Ir–O–Ir bond (3.01 Å) in IrO₂. The longer bond distance of Ir–O–Pt compared to Ir–O–Ir may result from the amorphous portion of IrPtO_x_ covering outside of the IrPt alloy. WT-EXAFS analysis further confirmed the differences between Ir–Pt and Ir–Ir, as well as between Ir–O–Pt and Ir–O–Ir (Fig. 3c). The WT-EXAFS at the Ir L_III_ edge exhibits two characteristic regions: a first shell domain for Ir–Pt scattering at R = 2.28 Å and K = 6.55 Å^−1^ and the second shell domain for Ir–O–Pt scattering at R = 3.21 Å and K = 4.45 Å^−1^. In contrast, Ir–Ir scattering in Ir foil occurs at R = 2.53 Å and K = 9.36 Å^−1^, while Ir–O–Ir scattering in IrO₂ occurs at R = 3.01 Å and K = 8.72 Å^−1^. The distinct K values confirm the formation of Ir–Pt bond and the long-range disordered Ir-O-Pt bonds in IrPtO_x_-S. The XANES spectrum at the Pt L_III_ edge indicates a predominantly metallic Pt surface with slight oxidation (Fig. 3d). The EXAFS spectrum shows two distinct peaks at 2.38 and 3.33 Å, corresponding to Pt–Ir and Pt–O–Ir scattering, respectively (Fig. 3e). The WT-EXAFS analysis, as shown in Fig. 3f, reveals the first shell domain for Pt–Ir scattering at R = 2.38 Å and K = 9.99 Å^−1^, distinct from Pt–Pt scattering in Pt foil (R = 2.51 Å, K = 10.03 Å^−1^), and the second shell domain for Pt–O–Ir scattering at R = 3.33 Å and K = 11.22 Å^−1^, distinguishable from scattering in Pt foil or PtO₂, confirming the different local atomic environment of Pt in IrPtOx-S from that of the references.

The Co K-edge XANES spectrum of IrPtOx-S resembles that of Co₃O₄ but with lower white line intensity, indicating reduced Co–O coordination due to doping-induced oxygen vacancies (Fig. S9a). The EXAFS spectrum exhibits Co₃O₄-like features but with lower peak intensity, consistent with the presence of oxygen vacancies (Fig. S9b). These findings align with XPS results, confirming the existence of oxygen vacancies, which facilitate electron transfer and improve catalytic activity [65]. The WT-EXAFS analysis at the Co K-edge confirms the different local atomic environment of Co in IrPtOx-S from that of pure Co₃O₄ (Fig. S10).

### Electrochemical Performance of IrPtOx-S

**Fig. 4:**
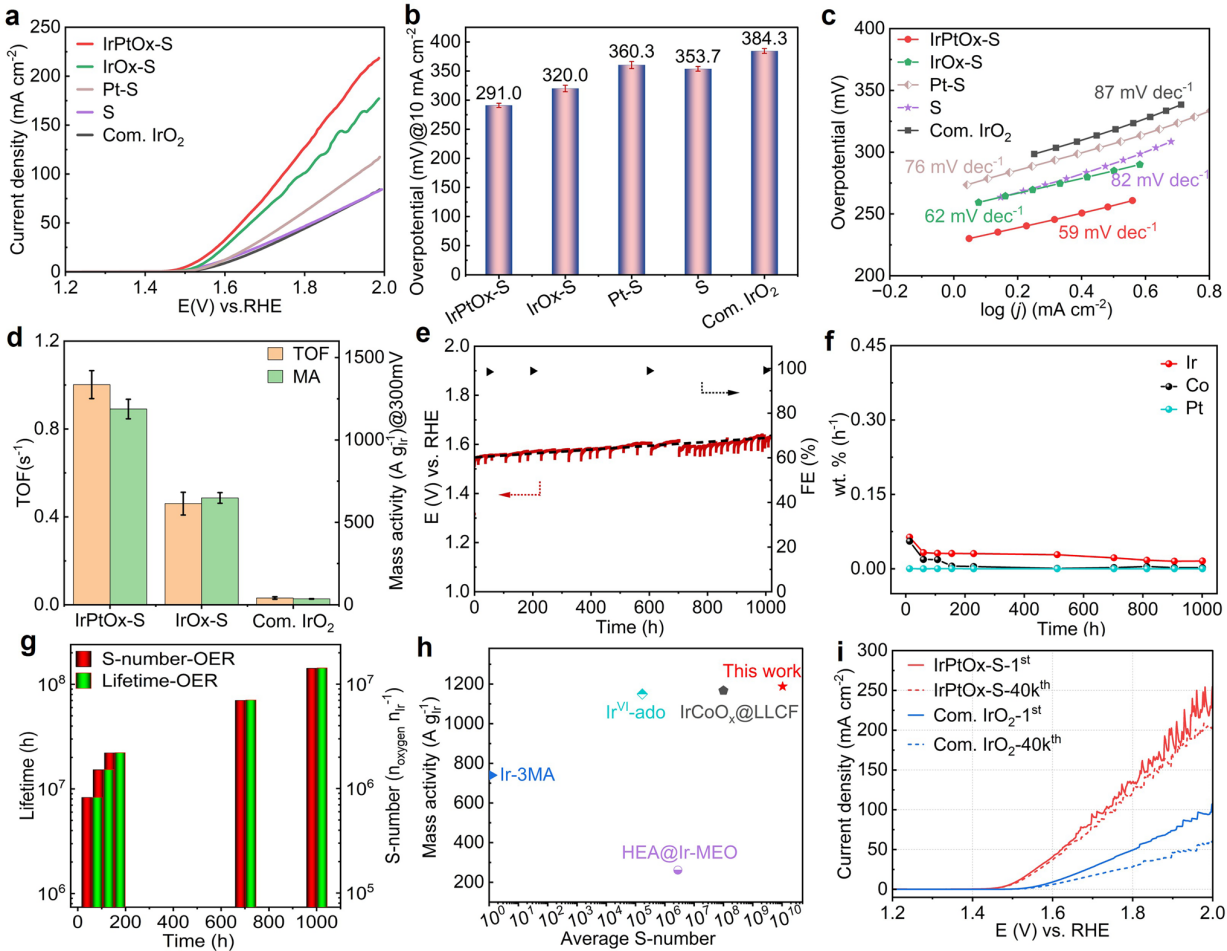
Electrochemical OER of IrPtOx-S. **a** Polarization curves recorded on different samples using a three-electrode configuration in O_2_-saturated 0.1 M HClO_4_ electrolyte. Ir/Pt loading for IrPtOx-S, IrOx-S, and Pt–S was 10 μg_Ir/Pt_ cm^−2^, respectively. Ir loading for Com. IrO_2_ was 44 ug_Ir_ cm^−2^. The loading of support material (S) was 150 μg cm^−2^. **b** overpotential at 10 mA cm^−2^ current density for different samples. **c** The relevant Tafel plots of the catalysts studied in (a). **d** Ir mass activities and TOF of different catalysts at an overpotential of 300 mV. **e** Chronoamperometric measurement on IrPtOx-S catalyst at 10 mA cm^−2^ current density for 1020 h in O_2_-saturated 0.1 m HClO_4_ electrolyte, and the FE as function of time. **f** The corresponding ICP-MS measurements and **g** S-number and lifetime calculation during chronoamperometric measurement shown in (**e**). **h** Comparison of S-number and Ir mass activity of IrPtOx-S to those of reported Ir-based OER catalysts.** i** Polarization curves of IrPtOx-S before and after 40,000 CV cycles from 1.3 to 1.8 V vs. RHE in O_2_-saturated 0.1 m HClO_4_ electrolyte. Com. IrO_2_ was the benchmark

The OER performance of IrPtOx-S was evaluated in O₂-saturated 0.1 M HClO₄ using a three-electrode cell. For comparison, support (S), Pt–S, IrO_x_-S, and Com. IrO₂ were tested under the same conditions, with all potentials referenced to RHE. As anticipated, IrPtO_x_-S demonstrated the highest catalytic activity among all tested materials (Fig. 4a). Notably, S outperformed Com. IrO₂ (with an Ir loading of 44 μg cm^−2^), underscoring the effectiveness of the support. The introduction of Pt improved the reaction kinetics of Pt–S at high potentials, while Ir significantly enhanced the OER activity of IrO_x_-S, reducing the overpotential to reach 10 mA cm^−2^ from 360.3 mV for Pt–S (10 μg_Pt_ cm^−2^) to 320.0 mV for IrOx-S (10 μg_Ir_ cm^−2^). By forming an Ir–Pt alloy, IrPtO_x_-S achieved an exceptionally low overpotential of 291.0 mV at 10 mA cm^−2^ with an ultralow Ir loading of 10 μg cm^−2^, highlighting the synergistic effect of Pt in optimizing the electronic configuration of Ir for enhanced OER activity. The overpotentials at 10 mA cm^−2^ for all catalysts are summarized in Fig. 4b.

The exceptional activity of IrPtOx-S is further evidenced by its Tafel slope of 59 mV dec^−1^ (Fig. 4c), significantly lower than those of IrO_x_-S (62 mV dec^−1^), Pt–S (76 mV dec^−1^), S (82 mV dec^−1^), and Com. IrO₂ (87 mV dec^−1^). This indicates improved electrocatalytic kinetics, which is attributed to the unique Ir–Pt alloy structure. The intrinsic catalytic activity was quantified using turnover frequency (TOF) and mass activity (MA) at 300 mV overpotential (Fig. 4d). The TOF of IrPtO_x_-S was 1.00 ± 0.06 s^−1^, 2.2 and 33 times higher than those of IrO_x_-S (0.46 ± 0.05 s^−1^) and Com. IrO₂ (0.03 ± 0.004 s^−1^), respectively. Similarly, the MA of IrPtO_x_-S reached 1188.0 ± 30 A g_Ir_^−1^, significantly surpassing those of IrO_x_-S (648.0 ± 32 A g_Ir_^−1^) and Com. IrO₂ (35.7 ± 2 A g_Ir_^−1^), further underscoring its superior performance.

The long-term stability of IrPtO_x_-S was evaluated via chronoamperometric (CA) at 10 mA cm^−2^ for 1020 h, demonstrating remarkable durability with only a 30 mV increase in potential (Fig. 4e). Gas chromatography (GC) confirmed the high purity of produced O₂ (> 99% faradaic efficiency, FE) throughout the test (Fig. 4e). ICP-MS revealed negligible dissolution of Ir, Pt, and Co during the CA test interval (Fig. 4f), highlighting the material’s exceptional stability under harsh acidic conditions.

The S-number (a metric for catalyst stability [47]) and lifetime of IrPtO_x_-S reached 1.42 × 10^7^ n_O2_ n_Ir_^−1^ and 1.43 × 10⁸ h (Fig. 4g), respectively, surpassing most reported Ir- and Ru-based OER catalysts in acidic media (Fig. 4h, Table S3). Accelerated stress tests (AST) involving 40,000 cycles from 1.3 to 1.8 V further confirmed the superior stability of IrPtO_x_-S compared to Com. IrO₂ (Fig. 4i), with only an 8 mV increase in potential at 10 mA cm^−2^ after the test. In contrast, the potential increased by 37 mV for Com. IrO₂ under the same conditions, underscoring the exceptional durability of IrPtO_x_-S. These results collectively demonstrate the outstanding OER activity and stability of IrPtO_x_-S in acidic media, positioning it as a leading candidate for practical PEMWE applications.

**Fig. 5:**
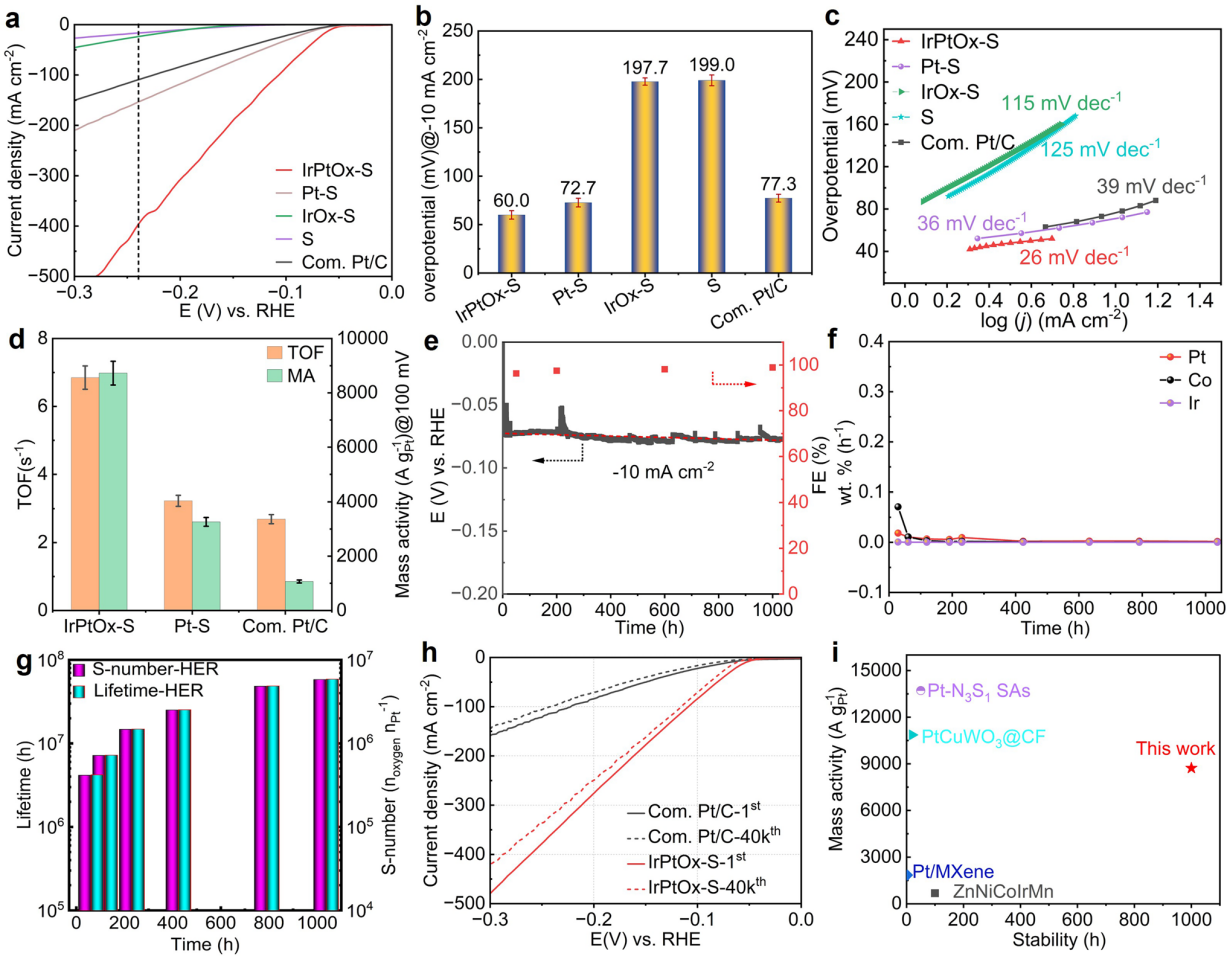
Electrochemical HER of IrPtOx-S. **a** Polarization curves recorded on different samples using a three-electrode configuration in H_2_-saturated 0.1 M HClO_4_ electrolyte. Pt/Ir loading for IrPtOx-S, IrOx-S, and Pt–S was 10 μg_Pt/Ir_ cm^−2^, respectively. The loading of support material (S) was 150 ug cm^−2^. Pt loading for Com. Pt/C was 20 ug_Pt_ cm^−2^. **b** overpotential at − 10 mA cm^−2^ current density for different samples. **c** The relevant Tafel plots of the catalysts studied in (**a**). **d** Pt mass activities and TOF of different catalysts at an overpotential of 100 mV. **e** Chronoamperometric measurement on IrPtOx-S at − 10 mA cm^−2^ current density for 1040 h in H_2_-saturated 0.5 M H_2_SO_4_ electrolyte, and the FE as function of time. **f** The corresponding ICP-MS measurements and **g** S-number and lifetime calculation during Chronoamperometric measurement shown in (**e**). **h** Polarization curves of IrPtO_x_-S before and after 40,000 CV cycles from 0.0 to − 0.8 V vs. RHE in H_2_-saturated 0.5 M H_2_SO_4_ electrolyte. **i** Comparison of S-number and Pt mass activity of IrPtO_x_-S to those of reported Pt-based and Ir-based HER catalysts

The HER performance of IrPtOx-S catalyst was evaluated in H₂-saturated 0.1 M HClO₄, with Pt–S, IrOx-S, S as control samples and Com. Pt/C as benchmark. Support (S) exhibited moderate HER activity, requiring an overpotential of 199.0 mV to reach − 10 mA cm^−2^, comparable to non-precious metal catalysts [67]. The introduction of Pt and Ir significantly improved the HER activity, with Pt–S outperforming Com. Pt/C (Fig. 5a). Specifically, Pt–S achieved − 10 mA cm^−2^ at overpotential of 72.7 mV with a Pt loading of 10 μg_Pt_ cm^−2^, while 77.3 mV was required for Pt/C to reach the same current density with a higher Pt loading of 20 μg cm^−2^ Pt. The IrPtO_x_-S catalyst demonstrated the highest HER activity, achieving − 10 mA cm^−2^ at overpotential of 60.0 mV and delivering a current density of 400 mA cm^−2^ at − 0.24 V, 2.6 and 3.7 times higher than that produced by Pt–S (− 153.7 mA cm^−2^) and Pt/C (− 108.7 mA cm^−2^) at the same potential (Fig. 5a). The overpotential at −10 mA cm^−2^ of different samples is summarized in Fig. 5b. The Tafel slope of IrPtO_x_-S catalyst was 26 mV dec^−1^, lower than those of Pt–S (36 mV dec^−1^), IrO_x_-S (115 mV dec^−1^), and Pt/C (39 mV dec^−1^) (Fig. 5c), indicating faster HER kinetics and a Tafel reaction pathway [45]. The TOF and MA of the IrPtO_x_-S catalyst at 100 mV overpotential were 6.85 ± 0.23 s^−1^ and 8725.0 ± 70 A g _Pt_^−1^, respectively, 2.1&2.6 and 2.5&8.2 times higher than those of Pt–S (3.23 ± 0.16 s^−1^, 3262.0 ± 50 A g _Pt_^−1^) and Pt/C (2.69 ± 0.13 s^−1^, 1069.0 ± 60 A g _Pt_^−1^) (Fig. 5d), respectively. These results highlight the exceptional intrinsic activity of the IrPtOx-S catalyst for HER.

Long-term stability was confirmed via CA testing at − 10 mA cm^−2^ for 1040 h, with only a 4 mV increase in potential at the end of test (Fig. 5e). GC and ICP-MS measurements confirmed high H₂ purity (> 99.0% FE) and almost negligible metal dissolution rates throughout the test (Fig. 5e, f). Furthermore, we measured the FE at various current densities for both HER and OER (Fig. S11). The results demonstrate that IrPtO_x_-S maintains high selectivity (> 98.6% FE) for both reactions even at elevated current densities. The S-number and lifetime for HER reached 5.8 × 10⁶ n_O2_ n_Ir_^−1^ and 6.0 × 10⁷ h, respectively (Fig. 5g), ranking the IrPtO_x_-S catalyst among the best HER catalysts reported [68]. AST involving 40,000 CV cycles scanning from 0 to − 0.8 V further demonstrated the high durability of IrPtO_x_-S, with only an 8 mV increase in overpotential at 10 mA cm^−2^ after testing (Fig. 5h), superior to that of Pt/C. The comparison of S-number and MA for HER of the IrPtOx-S catalyst to the reported Pt-based HER catalysts is shown in Fig. 5i and Table S3, underscoring its exceptional performance.

A comprehensive study of the ECSA of IrPtO_x_-S conducted via double-layer capacitance (C_dl_) measurements, H-UPD, and redox-active surface charge analysis reveals that IrPtOx-S exhibits a relatively higher ECSA compared to Com. IrO₂ and Pt/C (Figs. S12 and S13, Table S4). IrPtOx-S exhibited high specific activity for overall water splitting, confirming its robust intrinsic catalytic activity for both HER and OER (Figs. S14 and S15). Notably, compared to its OER-specific activity, IrPtO_x_-S displayed a more pronounced enhancement in HER-specific activity across all tested catalysts, including the benchmark. This highlights that the role of Ir in optimizing Pt’s catalytic activity for HER is more effective than Pt’s contribution to enhancing Ir’s activity for OER. We speculate that the incorporation of Pt into Ir significantly increased the ECSA of Ir species, potentially due to structural or electronic synergies between the two elements. The material’s hierarchical porosity, high surface area, large ECSA, fast kinetics, high concentration of oxygen vacancies, and intimate electronic interactions between Ir and Pt, combined with the intrinsic self-catalytic activity of the support (Fig. S16), collectively contribute to its exceptional bifunctional activity for OER and HER in acidic media.

### Performance of IrPtOx-S Under Realistic PEMWE Conditions

High catalytic activity observed in three-electrode systems (e.g., rotating disk electrode) does not necessarily correlate with performance in PEMWE, as the operating environments differ significantly in terms of electrode structure, temperature, pressure, and electrolyte. Therefore, evaluating catalysts under realistic PEMWE conditions is essential for practical applications. When operating under realistic PEMWE conditions, the hierarchical pore structure and electronic conductivity of the catalyst become critical for exposing active sites to reactants and electrolytes, managing water and product flow, and enabling fast electron transfer.

**Fig. 6:**
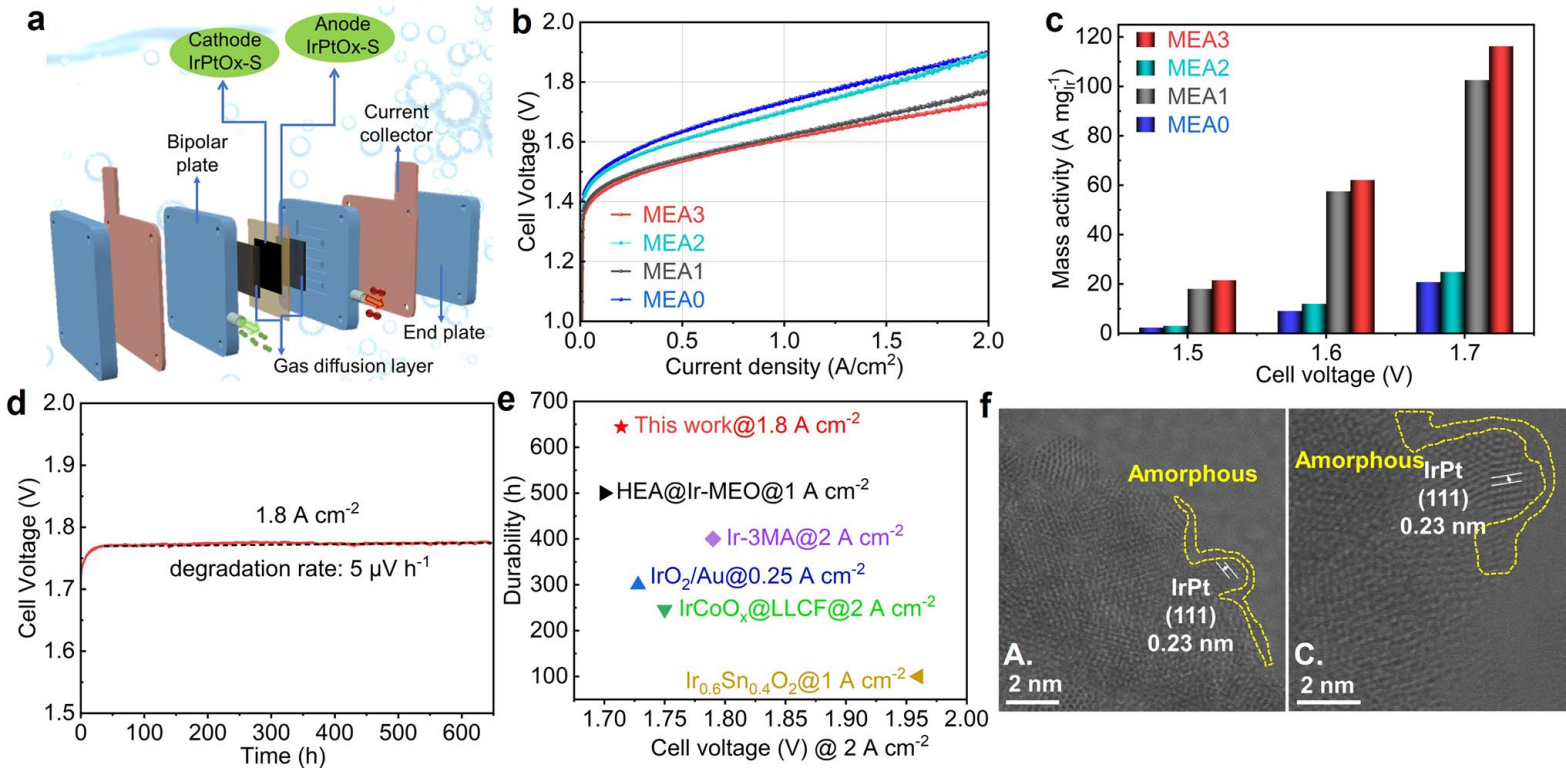
PEMWE performance: **a** Schematic of the PEMWE device we used for this study.** b** Polarization curves of different MEAs. **c** Comparison of Ir mass activity of MEA0-MEA3 at different selected voltages.** d** Long-term durability test of MEA3 at 1.8 A cm^−2^ current density with 0.075 mg_Ir_ cm^−2^ at anode and 0.075 mg_Pt_ cm^−2^ at cathode. **e** Comparison of Ir mass activity and cell durability of MEA3 to the reported ones. **f** TEM images of IrPtOx-S peeled off from MEA3 after long-term durability test. A. denotes anode, and C. denotes cathode

We fabricated the IrPtO_x_-S catalyst into a membrane electrode assembly (MEA) serving as both the anode (OER) and cathode (HER) (referred to as MEA3) and investigated its bifunctional performance in a PEMWE single cell at 80 °C and ambient pressure (Fig. 6a). To separately evaluate OER and HER activities, we prepared two additional MEAs: IrPtO_x_-S catalyst as the anode and Pt/C as the cathode (referred to as MEA1); and IrO₂/NbO_2_ as the anode and IrPtO_x_-S catalyst as the cathode (referred to as MEA2). A benchmark MEA0 was prepared using IrO₂/NbO_2_ as the anode and Pt/C as the cathode. For the commercial catalysts, e.g., IrO₂/NbO_2_ and Pt/C, the Ir and Pt loadings for the corresponding MEAs were set at 0.2 and 0.3 mg cm^−2^, respectively. For our catalyst, the Ir and Pt loadings were significantly reduced to 0.075 mg cm^−2^, highlighting its efficiency and cost-effectiveness.

As shown in Fig. 6b, MEA1 demonstrated superior performance compared to MEA0, achieving 1 A cm^−2^ at 1.63 V and 2 A cm^−2^ at 1.78 V. In contrast, MEA0 achieved 1 A cm^−2^ at 1.74 V and 2 A cm^−2^ at 1.91 V. This confirms the higher OER activity of IrPtO_x_-S compared to IrO₂/NbO_2_ under realistic PEMWE conditions. MEA2 showed slightly higher water splitting activity than MEA0, indirectly demonstrating the superior HER activity of the IrPtOx-S to that of Pt/C. Notably, the Pt loading in the cathode of MEA2 was only ¼ that of Pt in MEA0, further underscoring the efficiency of the IrPtO_x_-S. The performance difference between MEA1 and MEA0 was more pronounced than that between MEA2 and MEA0, as the overall PEMWE performance is predominantly determined by the OER activity at the anode.

MEA3, with IrPtO_x_-S at both electrodes, exhibited the best overall performance, achieving 1 A cm^−2^ at 1.60 V and 2 A cm^−2^ at 1.72 V. The Ir mass activity of MEA3 reached 116.3 A mg_Ir_^−1^ at 1.7 V, approximately 5.6 times higher than that of MEA0 (Fig. 6c). Additionally, MEA3 achieved an electrical efficiency of 72.2% at 2.0 A cm^−2^, with energy consumption of 43.1 kWh kg^−1^H₂ at 1 A cm^−2^ and 46.0 kWh kg^−1^H₂ at 2 A cm^−2^. In contrast, MEA0 consumed 46.6 kWh kg^−1^H₂ at 1 A cm^−2^ and 51.2 kWh kg^−1^H₂ at 2 A cm^−2^, corresponding to an efficiency of 64.7% at 2 A cm^−2^. In addition, at the same applied potential, e.g., 1.72 V, our MEA reduces the cost per kilowatt by over 70% compared to the leading commercial catalyst. For instance, our MEA3 achieves a cost of $7.85 kW^−1^, whereas MEA0 incurs a significantly higher cost of $25.19 kW^−1^. The high BET surface area of IrPtO_x_-S ensures sufficient exposure of active sites, directly enhancing catalytic activity. Furthermore, its hierarchical porosity, comprising interconnected micropores and mesopores, facilitates influx of water molecules to active sites and promotes efflux of generated H₂/O₂ gas, thereby minimizing diffusion limitations and accelerating reaction kinetics, consequently a rapid mass transport. The synergy between high surface area (maximizing active-site accessibility) and hierarchical porosity (optimizing mass transport) enables sustained high current density (e.g., 2 A cm^−2^ at 1.72 V, Fig. 6b), even at elevated potentials. Additionally, the oxygen vacancy-rich structure (Fig. 2d) enhances electronic conductivity, which improves charge transfer efficiency and further boosts catalytic activity. Collectively, those advantages are reflected in the lower and more stable cell impedance of PEMWE with MEA3 as compared to PEMWE with MEA0 (Fig. S17).

MEA3 was subjected to a 646-h durability test at 1.8 A cm^−2^, demonstrating exceptional stability with an average degradation rate of 5 μV h^−1^ (Fig. 6d). Based on the U.S. Department of Energy (DOE) protocol, which defines lifetime as the time until a 10% voltage loss from the beginning-of-life operation [10], MEA3 can operate at 1.8 A  m^−2^ for at least 34,360 h with ultralow loadings of 0.075 mg_Ir_ cm^−2^ at the anode and 0.075 mg_Pt_ cm^−2^ at the cathode. This exceptional performance positions the IrPtO_x_-S as a leading candidate for industrial PEMWE applications.

The performance metrics of IrPtOx-S, including lifetime and cell voltage at 2 A cm^−2^ current density under PEMWE conditions, were compared to those of reported catalysts (Fig. 6e, Table S3). MEA3 outperformed most Ir- and Pt-based MEAs, establishing itself as one of the best bifunctional catalysts for PEMWE (Fig. 6e). STEM-EDS characterization of IrPtOx-S peeled off from the anode and cathode of MEA3 after a 646-h durability test revealed no evidence of particle agglomeration or separation (Figs. S18 and S19). STEM-EDS line-scan profile (Fig. S18b, c) and HRTEM analysis (Fig. 6f) revealed no significant particle size change, aside from a minor increase in the thickness of IrPtO_x_ species, demonstrating exceptional structural stability under extended PEMWE operating conditions. XPS results revealed partial oxidation of surface Ir, as indicated by the increased proportion of Ir (IV) at the anode after the durability test (Fig. 2a), while negligible changes for Pt, Co, and O (Figs. 2b, c and S20). Raman spectroscopy further confirmed the structural integrity of the post-stability IrPtO_x_-S, with broader and weaker peaks (Fig. S21) indicating amorphization or reduced particle size, consistent with TEM observations (Fig. 6f).

### Mechanistic Insights into the Origin of Catalytic Activity

**Fig. 7:**
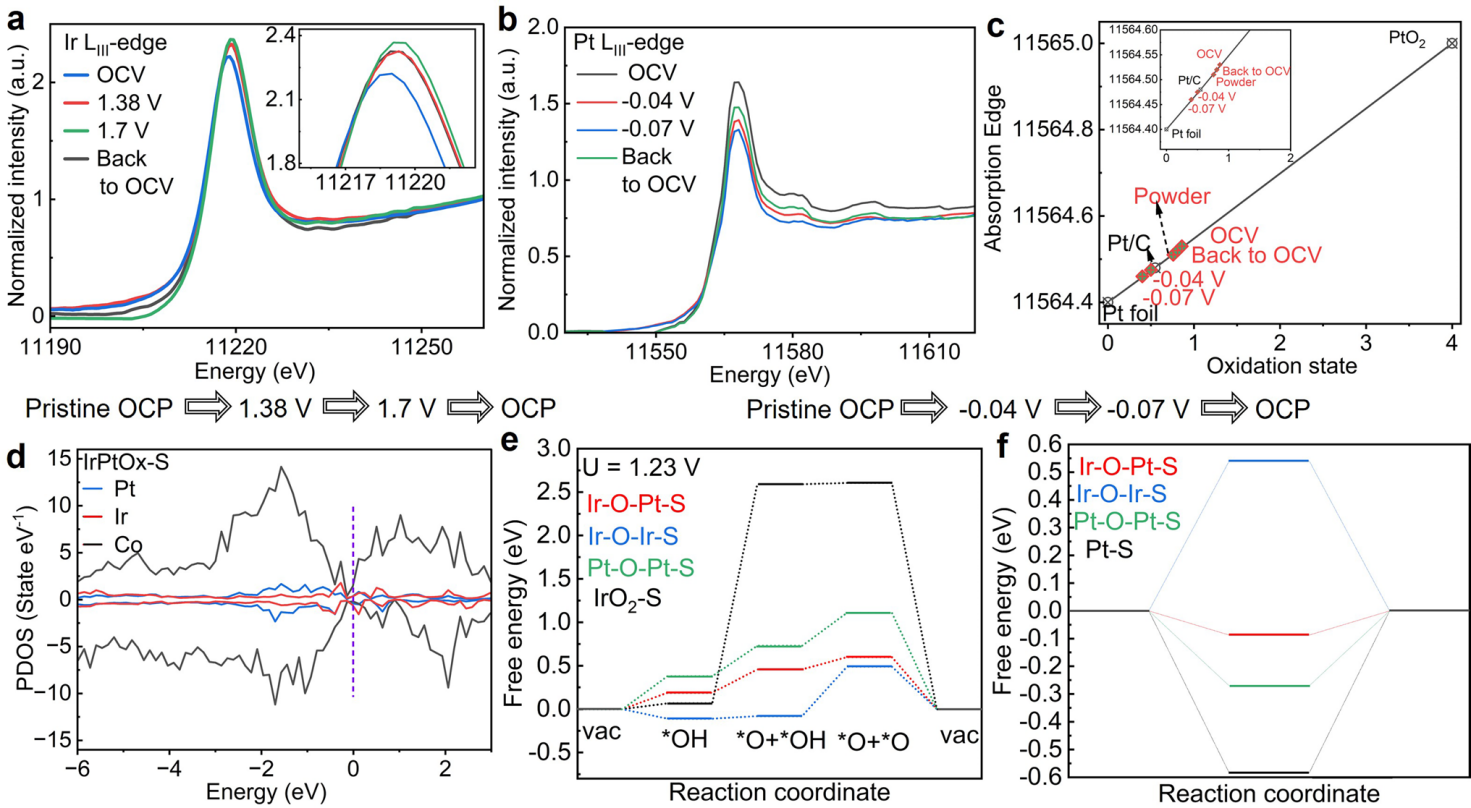
In situ XANES analysis and theoretical study of IrPtOx-S for OER and HER. **a** Ir L_III_ edge XANES measured in 0.1 M HClO_4_ during OER at OCV, 1.38V, 1.70 V, and reverse to OCV. Inset is the enlargement of the white line portion. **b** Pt L_III_ edge XANES measured in 0.5 M H_2_SO_4_ during HER at OCV, − 0.04V, − 0.07 V, and reverse to OCV. **c** Calculated Pt valence state based on the first differential of the absorption peak shown in **b.** Pt foil, Pt/C, and PtO_2_ were the references. **d** PDOS of Ir 5d, Pt 5d, and Co 3d of IrPtOx-S. **e** Gibbs free energy (ΔG_absorbates_*) diagrams of OER through bi-nuclear reaction mechanism for IrPtOx-S including Ir–O–Pt, Ir–O–Ir, and Pt–O–Pt sites and for IrO_2_-S. U = 1.23 V vs. RHE. **f** ΔG_H_* of HER at the equilibrium potential for IrPtOx-S including Ir–O–Pt, Ir–O–Ir, and Pt–O–Pt sites and for Pt–S

To investigate the active sites and monitor the evolution of chemical oxidation states during OER and HER, we performed in situ XANES and EXAFS experiments at the Ir L_III_, Pt L_III_, and Co K edges on IrPtOx-S. The sequence of applied potentials during OER and HER is illustrated in Fig. 7.

Under OER conditions, the WL position at the Ir L_III_ edge at open circuit potential (OCP) showed a slight positive shift compared to the IrPtOx powder, likely due to the adsorption of H₂O molecules in the electrolyte, causing electron delocalization (Figs. 3a and 7a). As the potential increased to 1.38 V, the WL position shifted to higher energy by 0.5 eV, accompanied by an increase in WL intensity (Fig. 7a, inset), indicating the oxidation of Ir sites. At 1.7 V, the WL peak position remained unchanged, but the intensity increased slightly, suggesting a slightly increased 5*d* hole states [69], a feature of higher activity. Compared to the dry sample (Fig. 3a), the Ir L_III_ WL peak positions at 1.38 and 1.70 V were close to that of IrO₂ reference, implying an increased fraction of Ir(IV) during the OER process. Previous studies have demonstrated that the formation of Ir(III) intermediates contributes to the instability of Ir-based catalysts [70]. The stability of IrPtO_x_-S is enhanced by the rapid conversion of Ir^0^ to Ir(IV). Given that XANES is a bulk-sensitive analytical technique, the white line energies represent average values of the oxidized iridium atoms in the shell and the metallic iridium atoms in the IrPt core. Therefore, the slight upshift of the WL energy upon the formation of the iridium oxide shell indicates that the contribution of the metallic bulk remains pronounced. When the potential reversed to OCP, the high oxidation state of Ir was retained (Fig. 7a, inset). Iridium in high oxidation states (e.g., Ir^4^⁺, Ir^5^⁺) exhibits strong electrophilic character, optimizing the binding strength of oxygen-containing intermediates (*OH, *O, *OOH) critical for oxygen evolution. Additionally, Fourier transform (FT) analysis of the Ir L_III_-edge EXAFS spectrum for the dry sample reveals an average Ir–Ir/Pt bond length of 2.75 Å (Figs. 3b and S22, Table S5), consistent with a configuration that facilitates direct O–O coupling (2.4–2.9 Å)—a bi-nuclear pathway [44]. By contrast, fitting IrO_2_ shows an Ir–Ir bond length of 3.22 Å. Therefore, promoting the oxidation of Ir to higher valence states may enhance both OER activity and durability of IrPtO_x_-S by enabling a bi-nuclear reaction pathway.

Under HER conditions, the Pt L_III_ edge WL intensity at OCP appeared enhanced compared to the IrPtOx-S powder (Figs. 3d and 7b), similar to the observation at the Ir L_III_ edge. This phenomenon is attributed to the adsorption of hydrogen (H_ads_), which increases the d-band hole count, reflected in the increased WL intensity [71]. As the applied potential became more negative, the WL intensity decreased (Fig. 7b), indicating the reduction of Pt to lower oxidation states. A similar trend was observed for PtW₆O₂₄/C during HER [72]. By plotting the differential of the absorption peak against the oxidation states of reference materials, the average oxidation state of Pt during HER was estimated to be around + 0.5 (− 0.04 V) and + 0.4 (− 0.07 V) (Fig. 7c), lower than that of Pt in the IrPtOx-S powder (Fig. 7c, inset), likely due to the reduction process during HER. The oxidation state of Pt reversed to a value comparable to that of Pt in the IrPtO_x_-S powder when the potential returned to OCP. Note that XANES represents the average values of the oxidation state of a given material, and based on our XPS analysis, the oxidation state of the oxidized Pt in the IrPtO_x_-S is approximately Pt(II), with metallic Pt dominating the IrPtO_x_-S. Pt(II) exhibits superior electrocatalytic activity for HER compared to Pt(0), as the Pt-O bond facilitates proton-electron coupling and accelerates H₂ release [72]. In summary, partial Ir and Pt in the Ir–Pt alloy and the amorphous regions evolved into relatively higher valence states and a stable structure, forming Ir-O-Pt bonds wrapped outside the IrPt nanoparticles during water splitting. These bonds serve as active sites for OER and HER, respectively.

In acidic media, HER proceeds via the Volmer–Heyrovsky or Volmer–Tafel mechanisms, distinguished by the Tafel slope. The Tafel slope of the IrPtO_x_-S for HER is 26 mV dec^−1^ (Fig. 5c), indicating that the Tafel step in the Volmer–Tafel mechanism is the rate-determining step [45]. For OER, three reaction pathways are possible: AEM, LOM, and oxide pair mechanism (OPM) [43]. To experimentally investigate the LOM pathway, we used in situ mass spectrometry (MS) with isotope labeling. IrPtOx-S was pretreated in 0.1 M HClO₄ with H₂^1^⁸O as the solvent to label the sample with ^1^⁸O. The sample was then used to catalyze water oxidation at 15 mA cm^−2^ for 30 min in 0.1 M HClO₄ with H₂^1^⁶O as the solvent (Fig. S23a). The ratios of ^3^⁶O₂/^32^O₂ in the produced oxygen were measured to be ~ 0.2% (Fig. S23b), similar to the natural abundance of ^1^⁸O [73]. In situ XANES spectra at the O K-edge showed negligible changes at OCP and under various applied potentials (Fig. S24), suggesting that lattice oxygen does not participate in the OER reaction. These results exclude the LOM mechanism.

To further understand the OER and HER mechanisms, we performed DFT calculations. The projected density of states (pDOS) of the Ir, Pt, and Co d-bands in IrPtO_x_-S revealed new hybridized electronic states and charge redistribution compared to IrOx-S and Pt–S (Figs. 7d and S25), indicating strong electronic interactions between Ir–Pt and the host. Specifically, the electron density of Pt in IrPtOx-S decreased, and the d-band center shifted negatively from − 0.094 eV (Pt–S) to − 2.438 eV (IrPtO_x_-S), weakening hydrogen chemisorption on Pt and enhancing HER kinetics [74]. Additionally, the pDOS showed increased electron density of Ir and Co near the Fermi level (≈ 0 to − 1 eV), suggesting more dangling bonds or active electronic states (Fig. S25). The d-band centers of Ir and Co in IrPtOx-S were upshifted (Ir: − 2.950 eV, Co: − 2.065 eV) relative to those in IrOx-S (Ir: − 3.866 eV, Co: − 2.398 eV), enhancing the interaction between active sites and adsorbates and reducing kinetic barriers for intermediate formation [69]. The overlapping Ir 5*d*, Pt 5*d*, and Co 3*d* orbitals further confirmed strong electronic interactions among these elements. Furthermore, Bader charge analysis reveals significant electronic interaction between Ir and Pt (Table S6), characterized by charge redistribution. This interaction balances the binding strength of key OER intermediates—mitigating over-binding at Ir–O–Ir sites and strengthening under-binding at Pt–O–Pt sites—thereby optimizing adsorption energetics and enhancing overall OER and HER activities.

The energetics of all intermediates (*OH, *O, *OOH, where * denotes an active site) during OER for each elementary step were calculated at an applied potential of 1.23 V vs. RHE (Fig. 7e). For the traditional AEM mechanism, there is a constant energy difference of 3.2 eV between *OH and *OOH that limits the exploration of more active electrocatalysts for water electrolysis. In this work, we adopt the bi-nuclear mechanism for OER, where the formation of problematic *OOH specie would be avoided, and a lower overpotential could be achieved [42]. Based on our experimental results, we employed Ir–O–Pt, Ir–O–Ir, and Pt–O–Pt configurations as the active sites for simulations, respectively, with IrO₂-S (OER) and Pt–S (HER) serving as benchmarks (Figs. S26 and S27). For OER on IrPtOx-S, the rate-determining step (RDS) is the first deprotonation of *OH to *O, exhibiting an exceptionally low activation energy barrier of 0.26 eV (Fig. 7e). In contrast, the RDS for OER on Ir-O-Ir shifts to the second deprotonation of *OH to *O, with the energy barrier increasing to 0.58 eV. Similarly, the RDS for OER on Pt–O–Pt is the same as that of Ir–O–Ir, but its energy barrier rises further to 0.67 eV. These results strongly suggest that Ir–O–Pt is the dominant active site, responsible for the superior OER activity of IrPtO_x_-S. Notably, the benchmark catalyst IrO₂-S shares the same RDS as Ir–O–Pt but displays the highest activation barrier (2.53 eV) among all studied catalysts. This stark contrast underscores the unique efficiency of the Ir–O–Pt configuration. Additionally, IrO₂-S might follow an AEM mechanism [29].

For HER, the adsorption free energy of H* (ΔGH*) is a key descriptor of activity in acidic media, with values closer to zero indicating higher activity [75]. IrPtO_x_-S showed significantly improved HER activity compared to Pt–S (Fig. 7f), highlighting the intimate interaction between Pt and Ir, as well as the importance of the Ir–O–Pt structure. As shown in Fig. 7f, ΔG_H_* for the Ir–O–Pt site (− 0.14 eV) was closer to the ideal (ΔGH* = 0 eV) compared to Ir–O–Ir (0.54 eV) and Pt–O–Pt (− 0.27 eV). This confirms that the Ir-O-Pt site is directly responsible for both optimizing hydrogen binding energetics and driving the exceptional HER activity of IrPtO_x_-S, as its near-ideal ΔG_H_* minimizes kinetic barriers during hydrogen evolution. Notably, the LaNi-Co₃O₄ (S) substrate itself exhibits inherent catalytic activity for both OER and HER, as confirmed by experimental characterizations and DFT simulations (Figs. S16 and S28). The in situ XANES characterizations combined with DFT simulations reveal that the exceptional bifunctional OER and HER performance of IrPtO_x_-S in acidic media arises from the catalytic synergy between IrPtO_x_ and substrate (S), and the optimized charge redistribution between Ir and Pt, which are bridged by O ligands, promoting the bi-nuclear and the Volmer–Tafel mechanism for OER and HER with reduced energy barriers. Additionally, the metal–support interaction along with the intimate affinity between Ir and Pt enables the formation of the unique IrPtO_x_ shell–IrPt core structure, expediting electron transfer for OER and HER to proceed.

## Conclusions

In this study, we developed a highly efficient and durable bifunctional catalyst, IrPtO_x_-S, for both OER and HER in acidic media. The catalyst demonstrated exceptional performance in PEMWE device, achieving a current density of 2 A cm^−2^ at 1.72 V with ultralow precious metal loadings (0.075 mg_Ir_ cm^−2^ Ir at the anode and 0.075 mg_Pt_ cm^−2^ Pt at the cathode). The IrPtOx-S exhibited remarkable stability, sustaining OER and HER for over 646 h in a PEMWE single cell with low average degradation rate of 5 μV h^−1^, outperforming most reported Ir- and Pt-based catalysts (Table S3).

Advanced characterization techniques, including in situ XANES, EXAFS, and DFT simulations, revealed that the high activity and stability of IrPtOx-S stem from the optimized charge redistribution between Ir and Pt, as well as the unique IrPt core–IrPtOx shell structure. The Ir sites and Pt sites bridged by O ligands, namely Ir–O–Pt sites, served as active sites promoting the bi-nuclear mechanism for OER and Volmer–Tafel mechanism for HER. The optimized charge redistribution and electronic structure reduced kinetic barriers for both OER and HER, while the unique metal core–oxide shell structure, abundant oxygen vacancies, hierarchical porosity, and high electrochemical surface area further enhanced electron and mass transfer, contributing to the catalyst’s superior performance.

This work not only provides a cost-effective solution for green hydrogen production but also offers fundamental insights into the design of high-performance bifunctional catalysts for PEMWE. The exceptional activity, durability, and industrial applicability of IrPtOx-S position it as a leading candidate for advancing PEMWE technology toward commercialization and achieving global decarbonization goals. By addressing the critical challenges of precious metal usage and catalyst stability, this study paves the way for the widespread adoption of PEMWE in renewable energy systems, marking a significant step toward achieving global decarbonization goals.

## Supplementary Information

Below is the link to the electronic supplementary material.

Supplementary file1 (DOCX 5779 KB)
