# Supplementary material for: Core–Shell IrPt Nanoalloy on La/Ni–Co3O4 for High-Performance Bifunctional PEM Electrolysis with Ultralow Noble Metal Loading
Source: Nanomicro Lett. 2025 Jul 14;17:329. doi: 10.1007/s40820-025-01845-7 (PMC12259513; doi:10.1007/s40820-025-01845-7)
Supplement: Supplementary file 1 — Supplementary file1 (DOCX 5779 KB) [file 40820_2025_1845_MOESM1_ESM.docx]

Supporting Information for

**Core-Shell IrPt Nanoalloy on La/Ni-Co_3_O_4_ for High-Performance Bifunctional PEM Electrolysis with Ultralow Noble Metal Loading**

Yifei Liu^1,2^, Xinmeng Er^3^, Xinyao Wang^4^, Hangxing Ren^1,2^, Wenchao Wang^1,2^, Feng Cao^1,2^, Taiyan Zhang^1,2^, Pan Liu^4^, Yakun Yuan^1,6^, Fangbo Yu^7^, Yang Ren^5^, Fuqiang Huang^1,2,^*, Wenjiang Ding^1,^* , Lina Chong^1,2,^*

^1^ Center of Hydrogen Science, School of Materials Science and Engineering, Shanghai Jiao Tong University, Shanghai 2000240, P. R. China

^2^ Zhangjiang Institute for Advanced Study (ZIAS), Shanghai Jiao Tong University, Shanghai 201210, P. R. China

^3^ State Key Laboratory for Mechanical Behavior of Materials, School of Materials Science and Engineering, Xi'an Jiao Tong University, Xi’an 710049, P. R. China

^4^ State Key Laboratory of Metal Matrix Composites, Shanghai Jiao Tong University, Shanghai 200240, P. R. China

^5^ Department of Physics, JC STEM Lab of Energy and Materials Physics, City University of Hong Kong, Hong Kong 999077, P. R. China

^6^ School of Mechanical Engineering, Shanghai Jiao Tong University, Shanghai 200240, P. R. China

^7^ International Research Center for Renewable Energy, State Key Laboratory of Multiphase Flow in Power Engineering, Xi’an Jiao Tong University, Shaanxi 710049, P. R. China

*Corresponding authors. E-mail: [chonglina@sjtu.edu.cn](mailto:chonglina@sjtu.edu.cn) (Lina Chong); [huangfq@sjtu.edu.cn](mailto:huangfq@sjtu.edu.cn) (Fuqiang Huang); [wjding@sjtu.edu.cn](mailto:wjding@sjtu.edu.cn) (Wenjiang Ding)

**Materials**

The chemicals including cobalt (II) nitrate hexahydrate (Co(NO_3_)_2_·6H_2_O , reagent grade, 98%), iridium(III) chloride hydrate (IrCl_3_·nH_2_O, 99.9%) and iridium black (Ir) were purchased from Sigma-Aldrich (American). The chemicals including La(NO_3_)_3_·xH_2_O (99.99% metals basis), Ni(NO_3_)_2_·6H_2_O (98%, AR), methanol (CH_3_OH, ≥ 99.9 %, GC), 2-methylimidazole (C_4_H_6_N_2_, ≥ 98%), benzyl alcohol(C_7_H_8_O,≥99%, AR), citric acid (C₆H₈O₇,≥99.5%(T), AR), iridium oxide (IrO_2_, 99.9% trace metals basis), ethanol (C_2_H_5_OH ≥99.8%), isopropanol (IPA, C_3_H_7_OH, GC, ≥ 99.9%) were purchased from Aladdin (China). 1,4-Phthalaldehyde (C_8_H_6_O_2_, ≥ 99%, GC) was purchased from Macklin (China). Platinum bis(acetylacetonate) (C_10_H_14_O_4_Pt, Pt 50%) was purchased from Kunming Guiyan New Material Technology Co., Ltd. (China) Acetone (C_3_H_6_O, ≥99.5%, AR) was purchased from Sinopharm. 5% Nafion solution (D520CS) were purchased from Chemours (American). Iridium oxide (75 wt%) on niobium oxide (IrO_2_ on NbO_x_) and 20 wt.% Pt on carbon black (Pt/C) were purchased from Umicore (British). Carbon was purchased from XFNANO (China). The HClO_4_ was purchased from thermo fisher scientific Chemicals (American). DI water with a resistance of ~18.25 MΩ·cm was produced by a pure water machine bought from Aikepu Company (China). All the chemicals were used directly without further purification.

**Supplementary Figures and Tables**

**
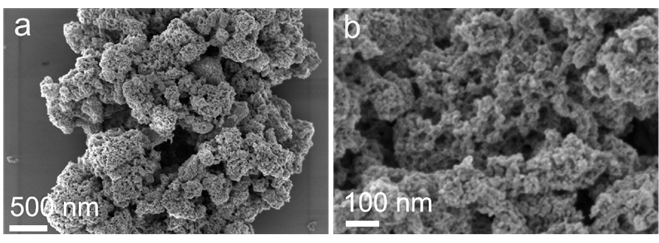
**

**Fig. S1** SEM images of IrPtOx-S. The results reveal a hierarchical pore structure composed of interconnected macropores and mesopores. This architecture facilitates efficient mass transport by enabling rapid influx of reactants (e.g., water) and efflux of products (H₂/O₂ gas)


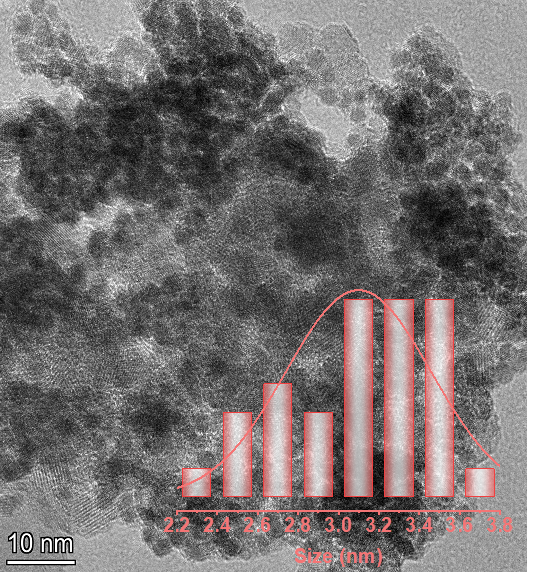


**Fig. S2** TEM images of IrPtOx-S and the corresponding particle size distribution of the entire catalyst


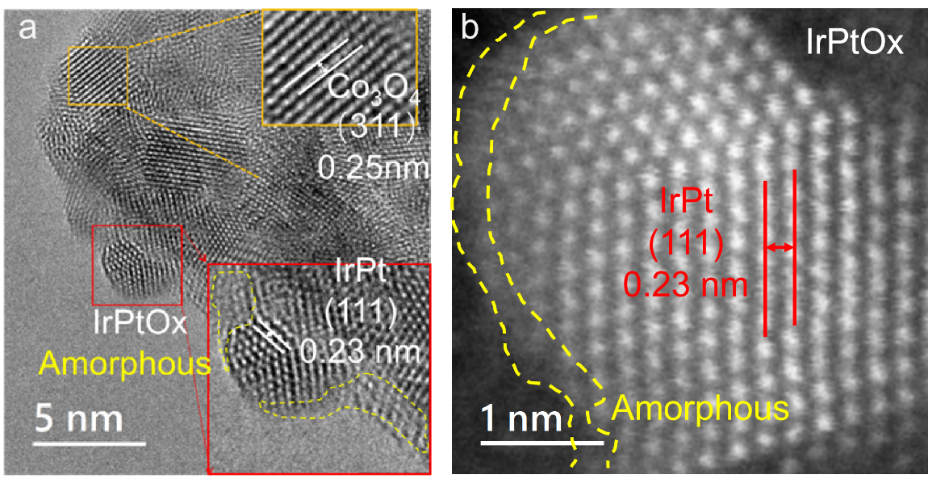


**Fig. S3** **a** HRTEM image of IrPtOx-S; **b** HRTEM image of IrPtOx single particle. It clearly reveals an amorphous phase with thickness of 1-2 nm surrounding the IrPt nanoparticles


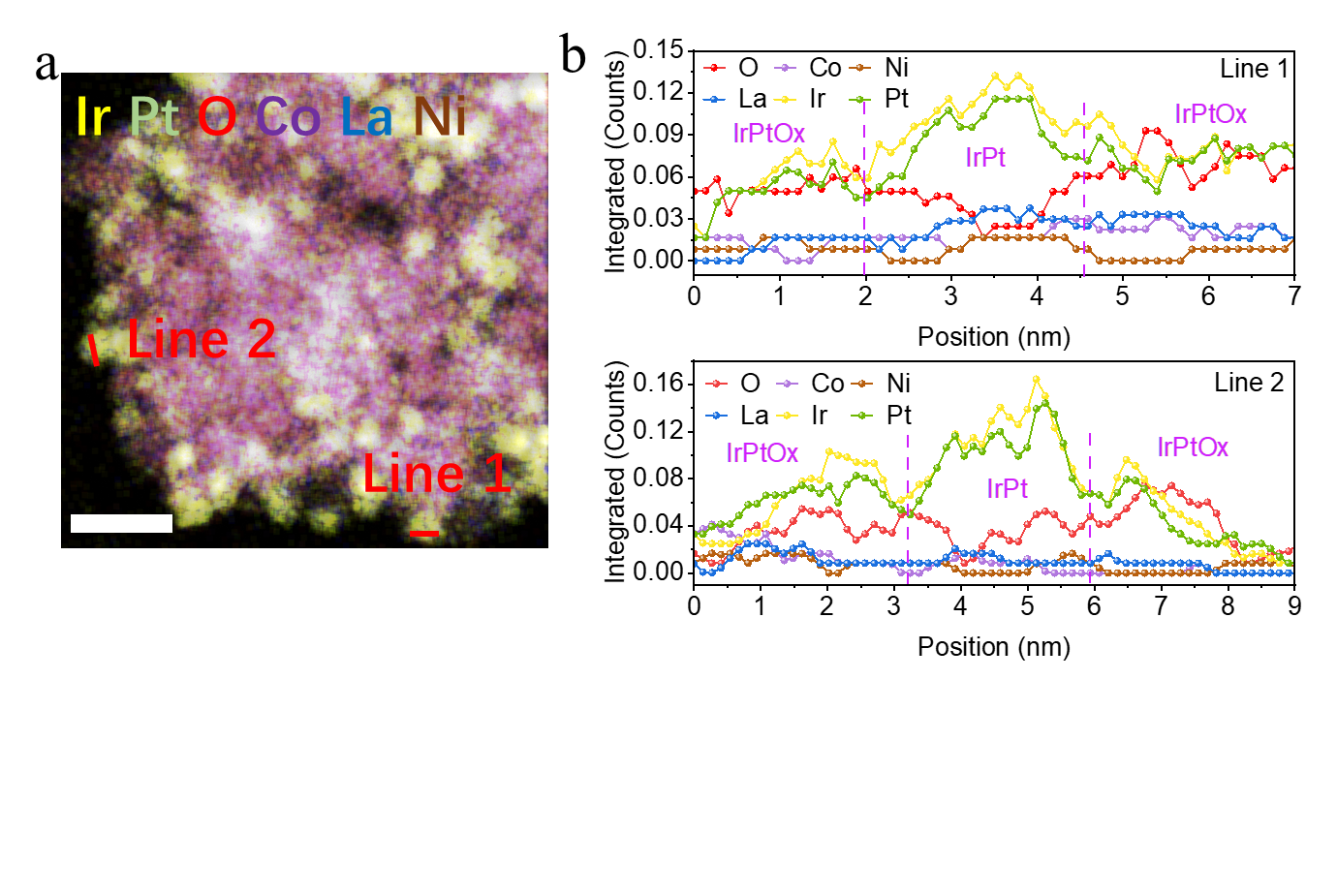


**Fig. S4** **a** STEM-HAADF -Mapping of IrPtOx-S; **b** line scan of two individual IrPtOx particles. The results show that Ir and Pt are in the form of alloy, and Co, O, La and Ni are uniformly distributed across the entire catalyst. STEM-EDS Line scan profiles further confirm the formation of IrPt alloy core with particle size being around 2-3 nm, and IrPtOx shell with thickness of 2-3 nm. Those structural observations align with our XPS and Raman analyses, detecting the formation of oxidized Ir and Pt species


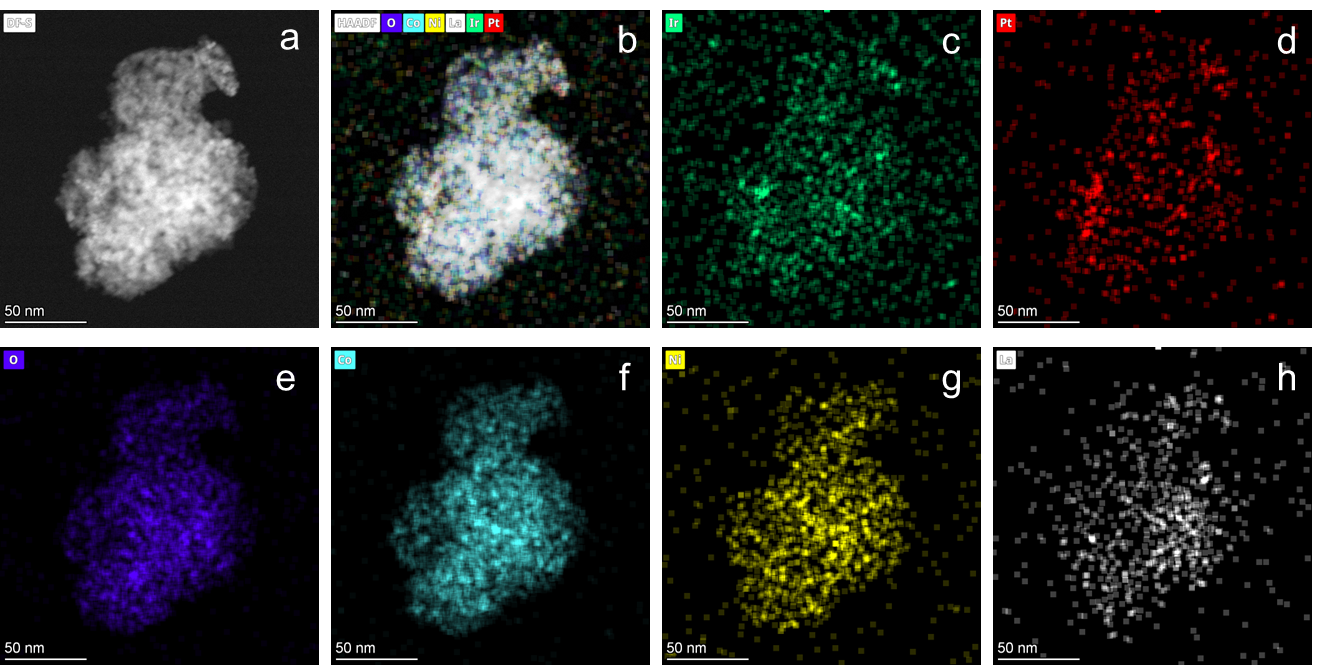


**Fig. S5** HAADF-STEM image of IrPtOx-S and elemental mappings. It reveals that Ir, Pt, Co, Ni, La, and O are uniformly distributed, with Ir and Pt primarily located on the surface of the support material


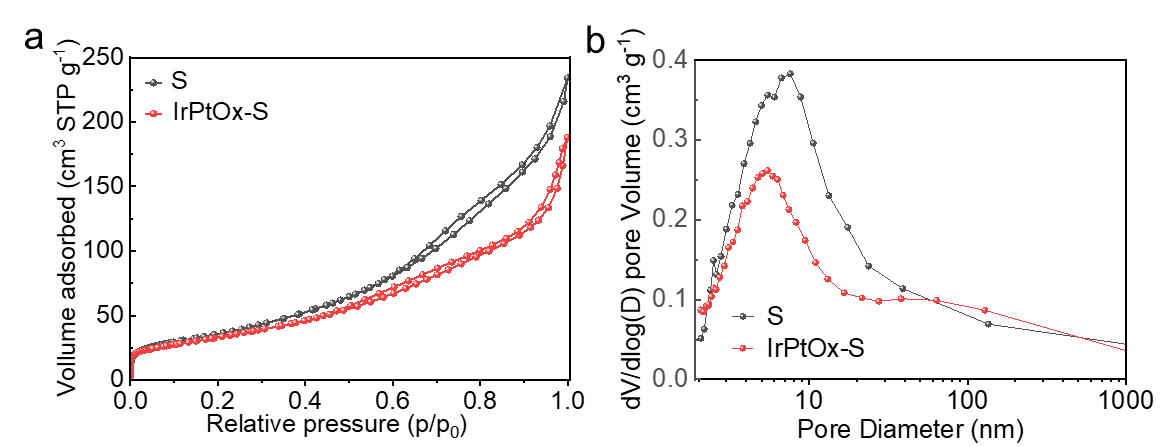


**Fig. S6** **a** N_2_ adsorption/desorption isotherms of the support material (S) and IrPtOx-S. **b** the corresponding pore size distribution


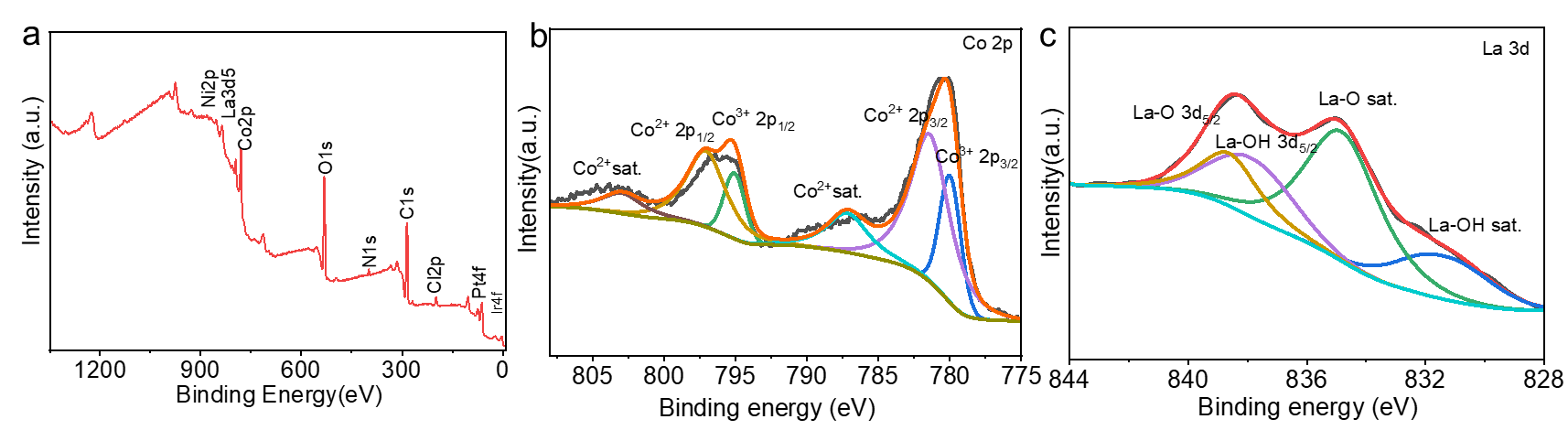


**Fig. S7** **a** XPS survey data of IrPtOx-S. **b** XPS spectra of Co 2p. **c** XPS spectra of La 3d. The ratio of Co²⁺ to Co³⁺ is significantly greater than 1:2, which is the ratio in pure Co_3_O_4_, indicating a high concentration of Co²⁺ in the support material. This suggests a high concentration of oxygen vacancies, consistent with the XPS results at O 1s


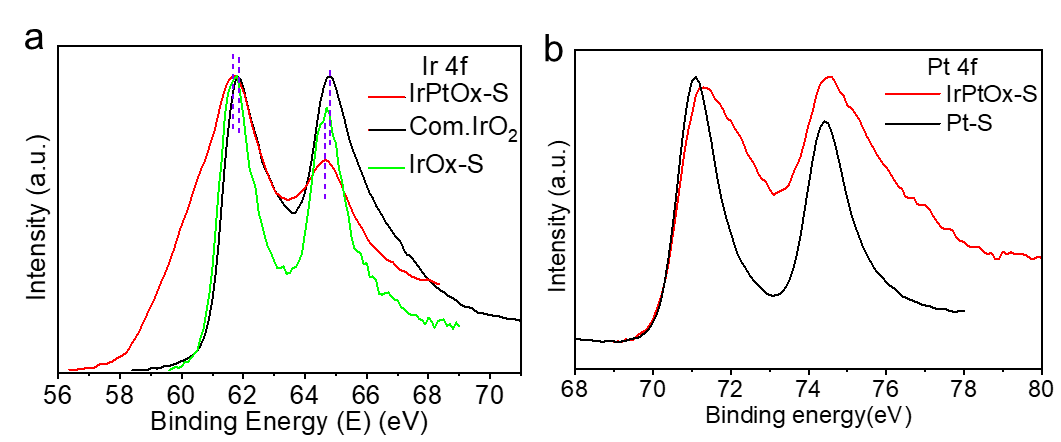


**Fig. S8** **a** XPS spectra of Ir 4f for IrPtOx-S, commercial IrO₂ (Com. IrO₂), and IrOx-S. **b** XPS spectra of Pt 4f for IrPtOx-S and Pt-S. The results indicate that the introduction of Pt facilitates the formation of metallic Ir, leading to the formation of IrPt alloy. Simultaneously, Pt particles were oxidized in the presence of Ir. These findings further confirm the formation of an Ir-O-Pt shell surrounding the IrPt alloy


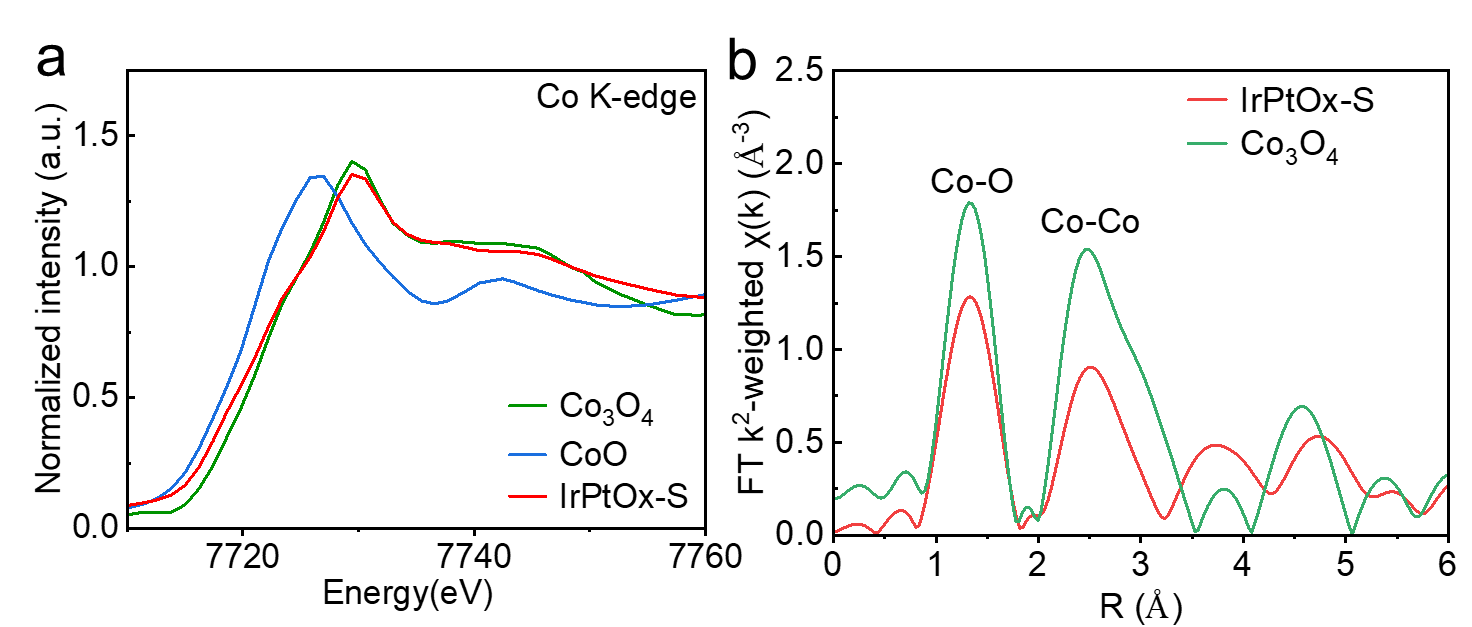


**Fig. S9 a** XANES and **b** EXAFS spectra of Co at K-edge of IrPtOx-S. Co_3_O_4_, CoO were the references


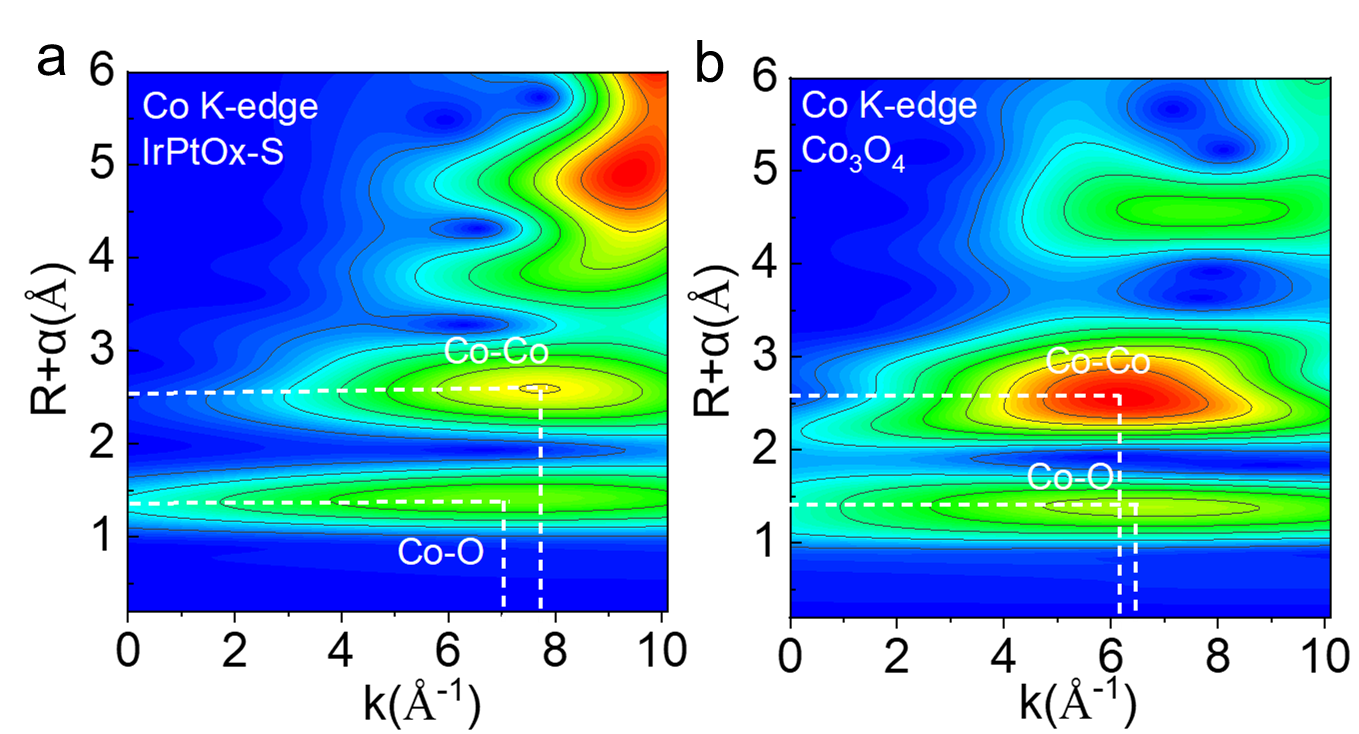


**Fig. S10** Wavelet transform images of Co K-edge EXAFS of pristine IrPtOx-S. Co_3_O_4_ was the reference. Obviously, the local atomic structure of Co as the support material for IrPtOx is different from that of pure Co_3_O_4_


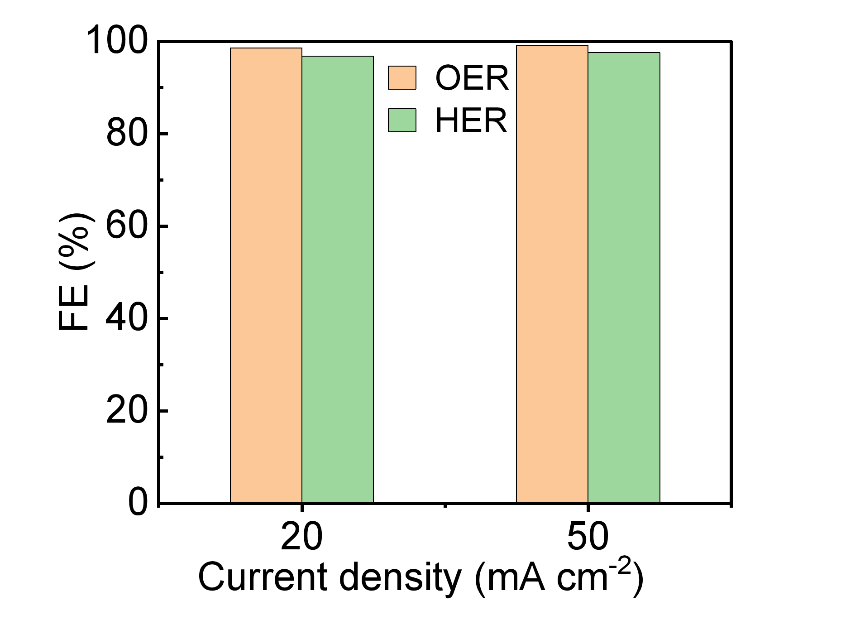


**Fig. S11** Faradaic efficiency (FE) of IrPtOx-S measured at different current densities of 20 and 50 mA cm⁻² during OER and - 20 and - 50 mA cm⁻² during HER processes in a three-electrode configuration using a 0.1 M HClO₄ aqueous electrolyte


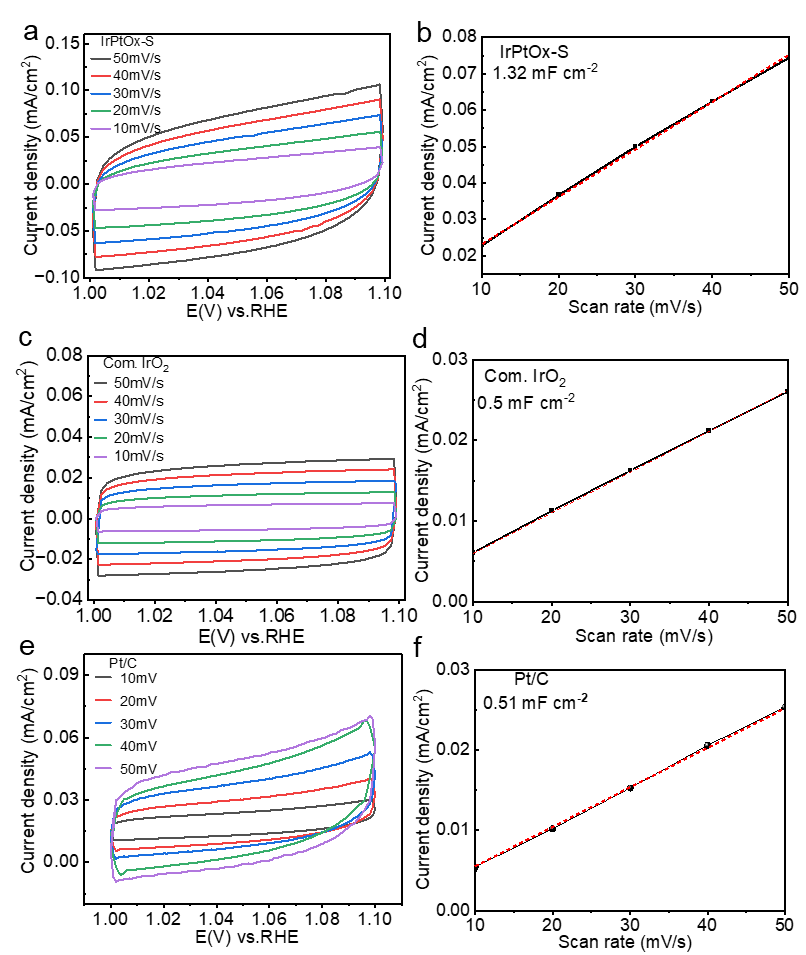


**Fig. S12** Typical cyclic voltammetry curves of IrPtOx-S **a** Com. IrO_2_ **c** and Com. Pt/C **e** at different scan rates of 10, 20, 30, 40, 50 mV s^-1^. The scanning potential range is from 1.0 V to 1.1 V where no faradic reaction takes place. The corresponding current density plotted against scan rates of different samples were shown in **b**, **d**, **f**


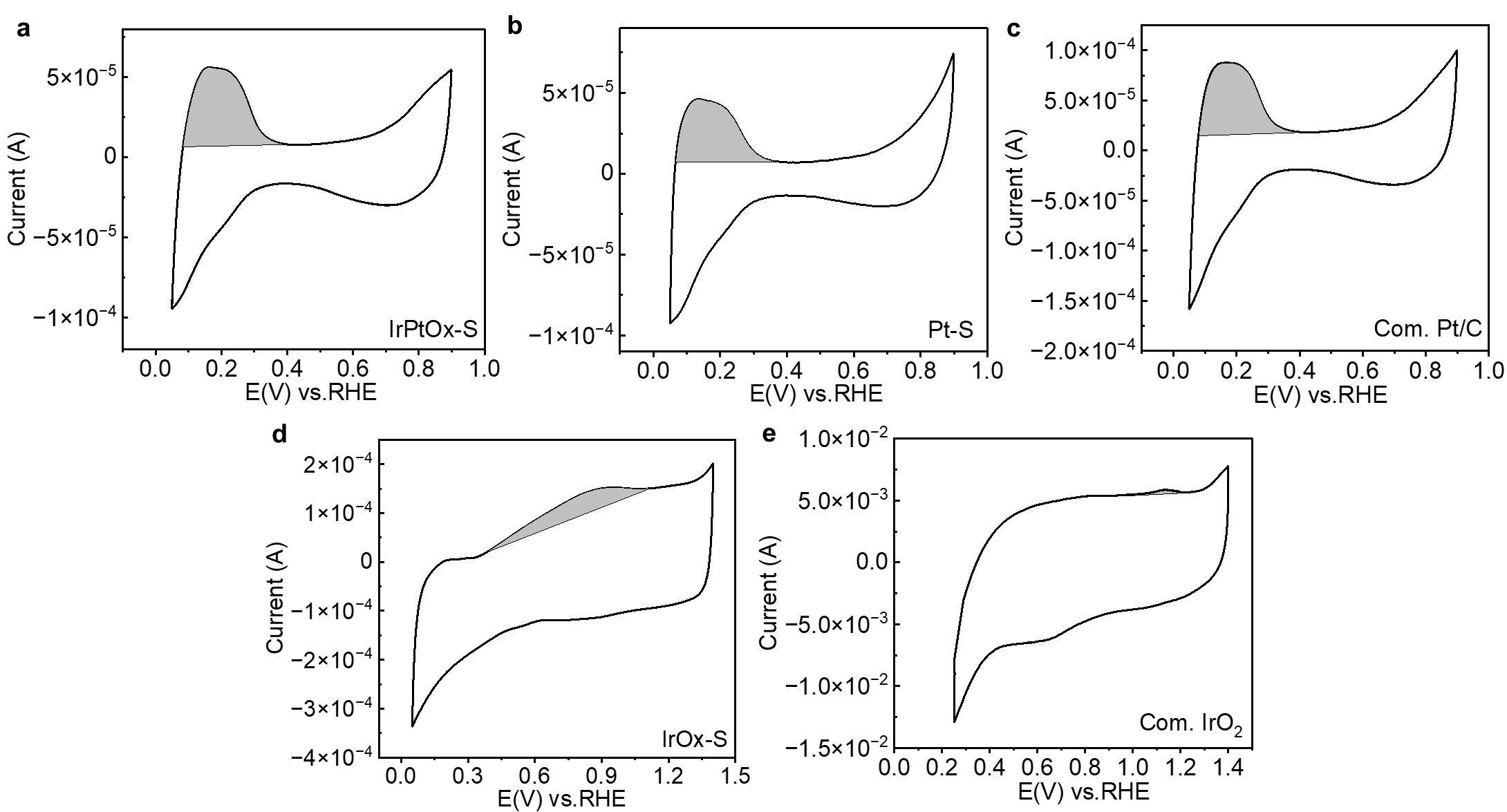


**Fig. S13** **a, b, c** ECSA of IrPtOx-S, Pt-S and the commercial Pt/C obtained by using Hydrogen Underpotential Deposition (H-UPD) method. **d, e** ECSA of IrOx-S and commercial IrO_2_ obtained by using Redox-Active Surface Charge (Redox Peaks) method. The ECSA values were shown in Table S4

**Fig. S14** OER specific activities of different catalysts, calculated by normalizing the polarization curve current to the ECSA of each material. It reveals that IrOx-S and IrPtOx-S exhibit comparable intrinsic activities. This suggests that the enhanced geometric activity of IrPtOx-S relative to IrOx-S likely arises from its larger ECSA, a result of incorporating Pt into the structure

**Fig. S15** HER specific activities of different catalysts, calculated by normalizing the polarization curve current to the ECSA of each material. The result suggests that incorporating iridium into platinum to form a surface with oxidized species significantly enhances the catalytic activity toward HER


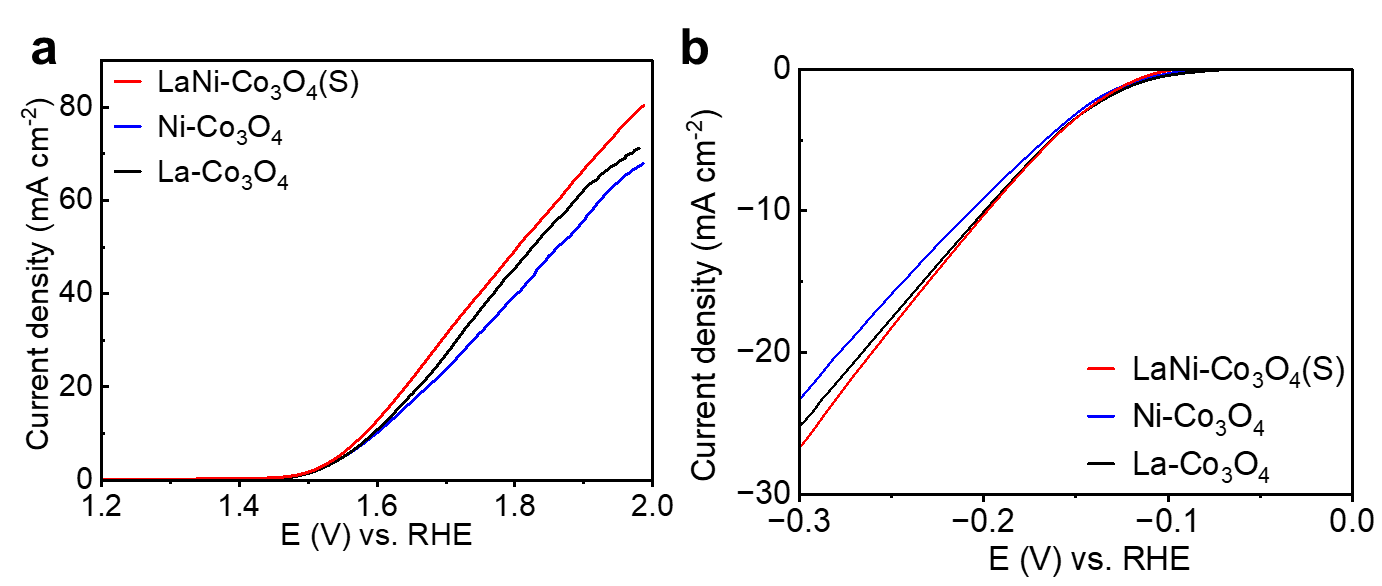


**Fig. S16** **a** Polarization curves recorded on LaNi-Co_3_O_4_ (S), Ni-Co_3_O_4_, La-Co_3_O_4_ for OER in O_2_ saturated 0.1 M HClO_4_ electrolyte. **b** Polarization curves recorded on LaNi-Co_3_O_4_ (S), Ni-Co_3_O_4_, La-Co_3_O_4_ for HER in O_2_ saturated 0.1 M HClO_4_ electrolyte. The loading of each material was 150 μg cm^-2^, the scan rate was 2 mV s^-1^


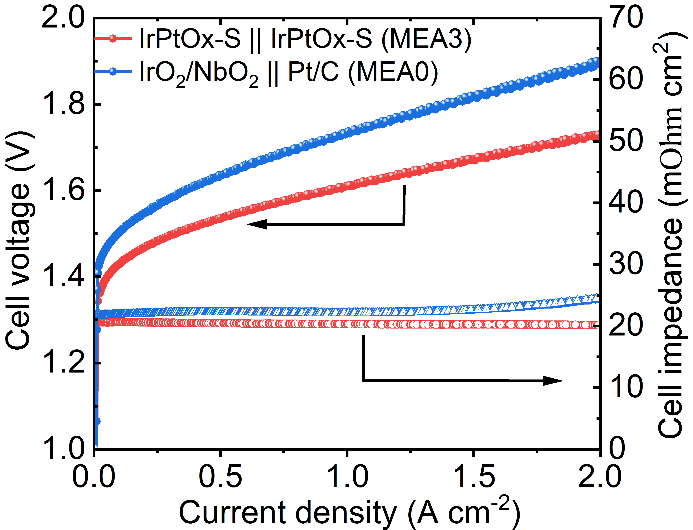


**Fig. S17** PEMWE cell polarization and impedance curves at 80 ^o^C as function of current density for IrPtOx-S and commercial IrO_2_/NbO_2_. Our IrPtOₓ-S catalyst exhibits a cell impedance constantly at ~ 20 mΩ·cm², outperforming the commercial benchmark (20 - 25 mΩ·cm²). This lower and stable impedance reflect enhanced electronic conductivity from metallic Ir/Pt cores, efficient charge transfer at Ir-O-Pt active sites, optimized mass transport due to hierarchical porosity. The results demonstrate the excellent integration of our catalyst into the MEA


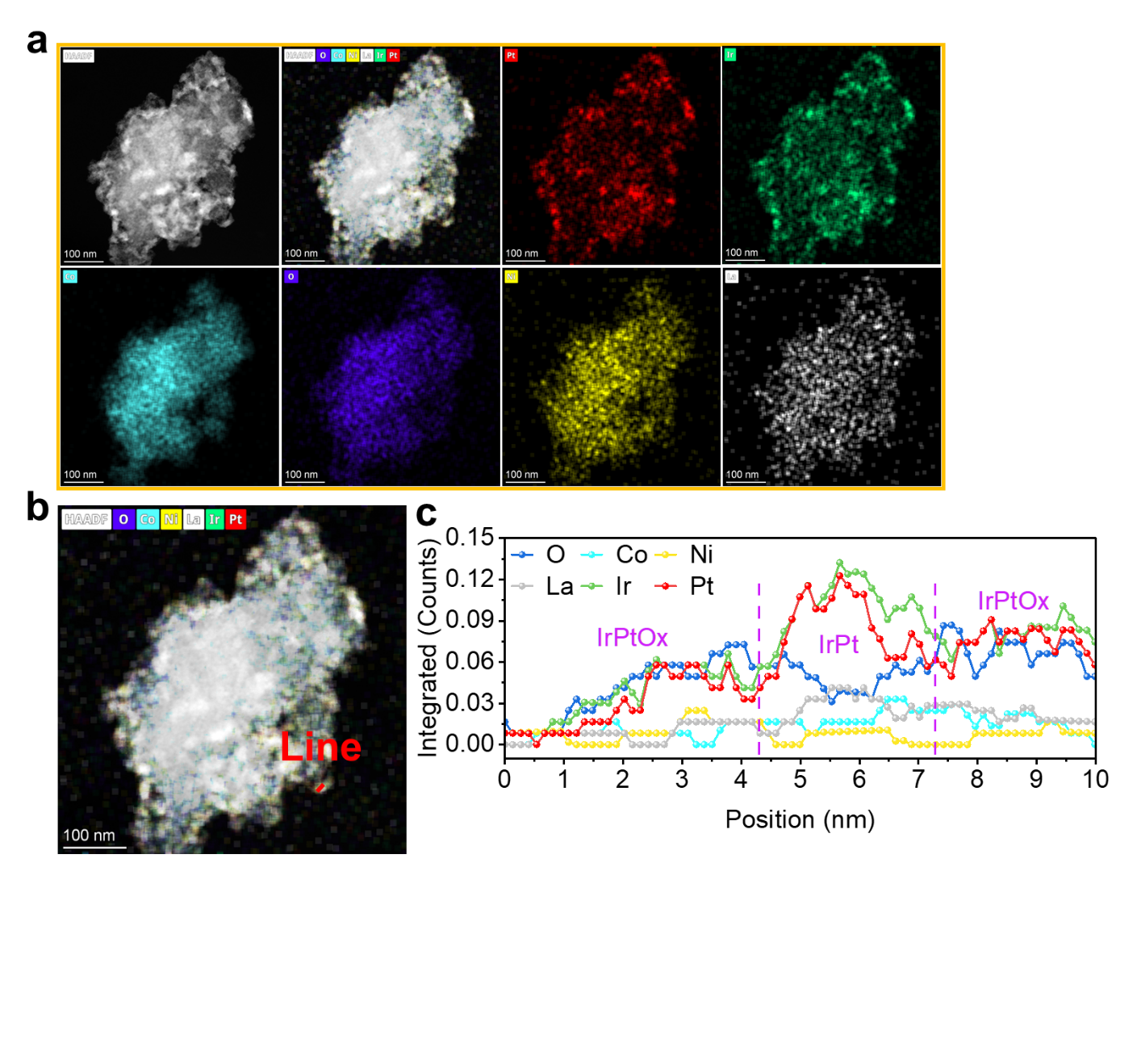


**Fig. S18** **a, b** HAADF-STEM-EDS images of IrPtOx-S peeled off from the anode of the MEA after 646 hours’ stability test. **c** Elemental line-scan profile across a single IrPtOx nanoparticle (NP), revealing compositional distribution


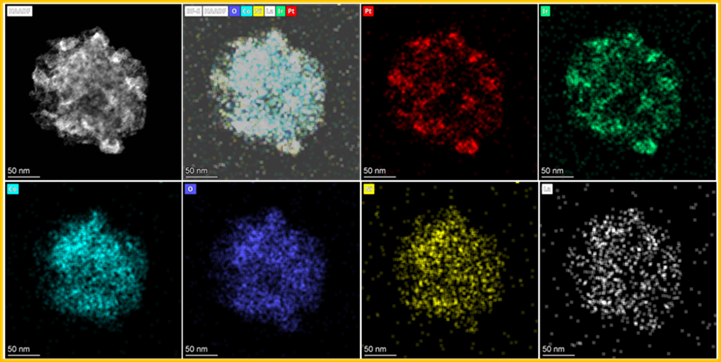


**Fig. S19** HAADF-STEM-EDS images of IrPtOx-S peeled off from the cathode of the MEA after 646 hours’ stability test

Figures S18 and S19 confirm that there is no particle agglomeration or separation/delamination, except only a minor increase in the IrPtOx layer thickness (approximately ~1 nm), consistent with post-test XPS and Raman analyses. The result underscores the structural and chemical robustness of the catalyst under prolonged electrochemical operation.


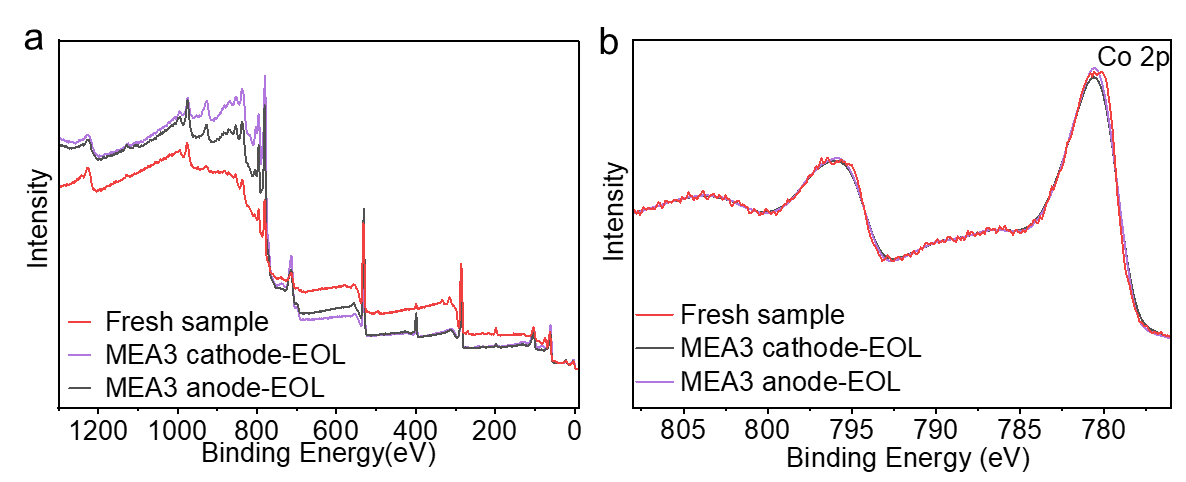


**Fig. S20** **a** XPS survey data of IrPtOx-S at the beginning of life (fresh sample) and the end of life (EOL) during durability testing in MEA3, including the anode and cathode. **b** XPS spectra of Co 2p for the fresh sample and the samples at EOL, including the anode and cathode

**Fig. S21** Raman spectra of the fresh IrPtOx-S, and the IrPtOx-S samples peeled off from the MEA after 644 hours testing


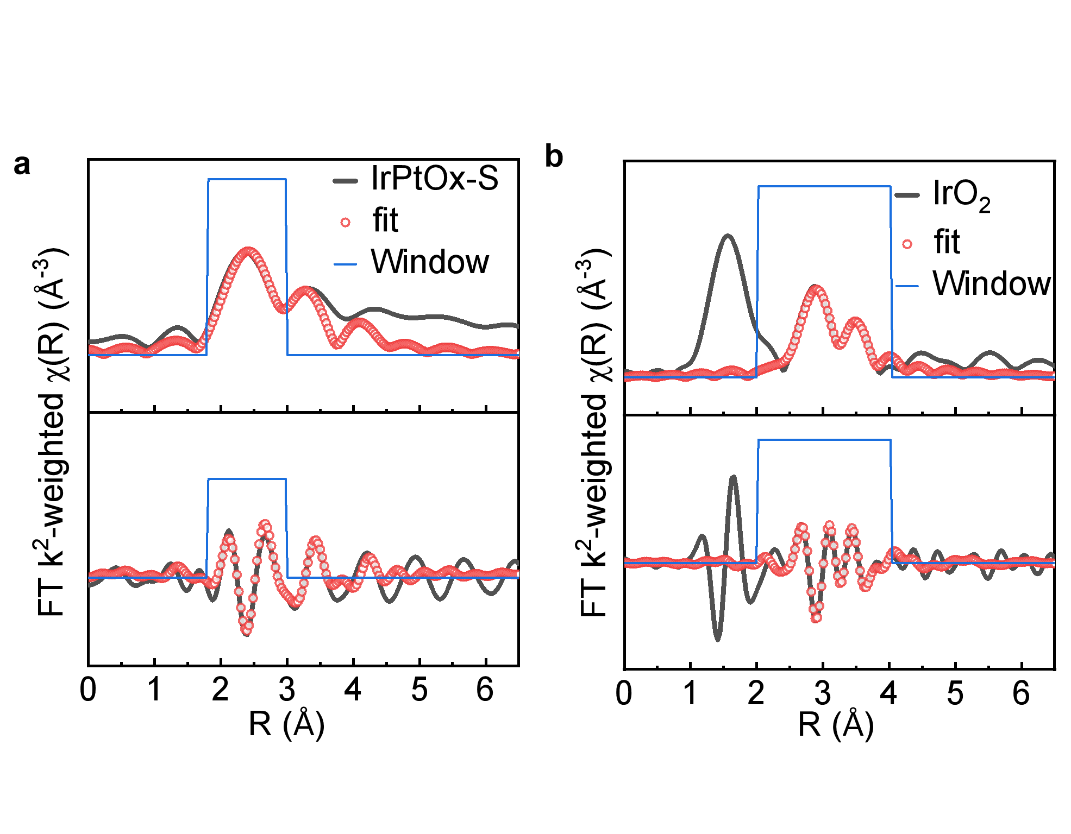


**Fig. S22** k^2^-weighted Fourier-transformed (FT) EXAFS spectrum collated at Ir L_III_-edge of IrPtOx-S and the fit. **a** IrPtOx-S; **b** IrO_2_


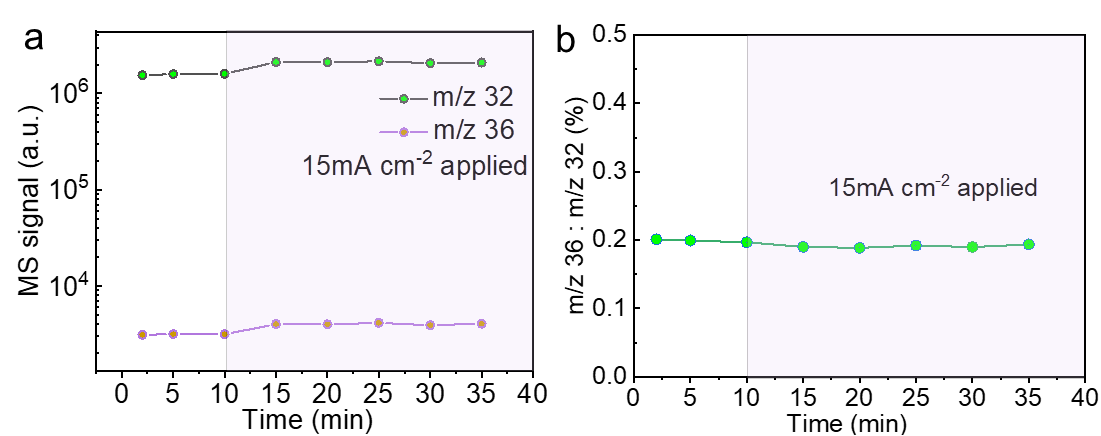


**Fig. S23** MS measurements of IrPtOx-S using a galvanostatic method. **a** MS signals of ^32^O_2_ (^16^O + ^16^O) and ^36^O_2_ (^18^O + ^18^O) collected during the galvanostatic test at 15 mA cm^-2^. **b** The ratio of ^36^O_2_: ^32^O_2_, which almost constant at ~ 0.2 % during 30 mins testing at 15 mA cm^-2^, suggesting ^36^O_2_ came from background ^18^O

**Fig. S24** XANES spectra at O k-edge measured at different potentials (OCV, 1.38 V, 1.70 V and reverse to OCV) in 0.1 M HClO_4_ during OER


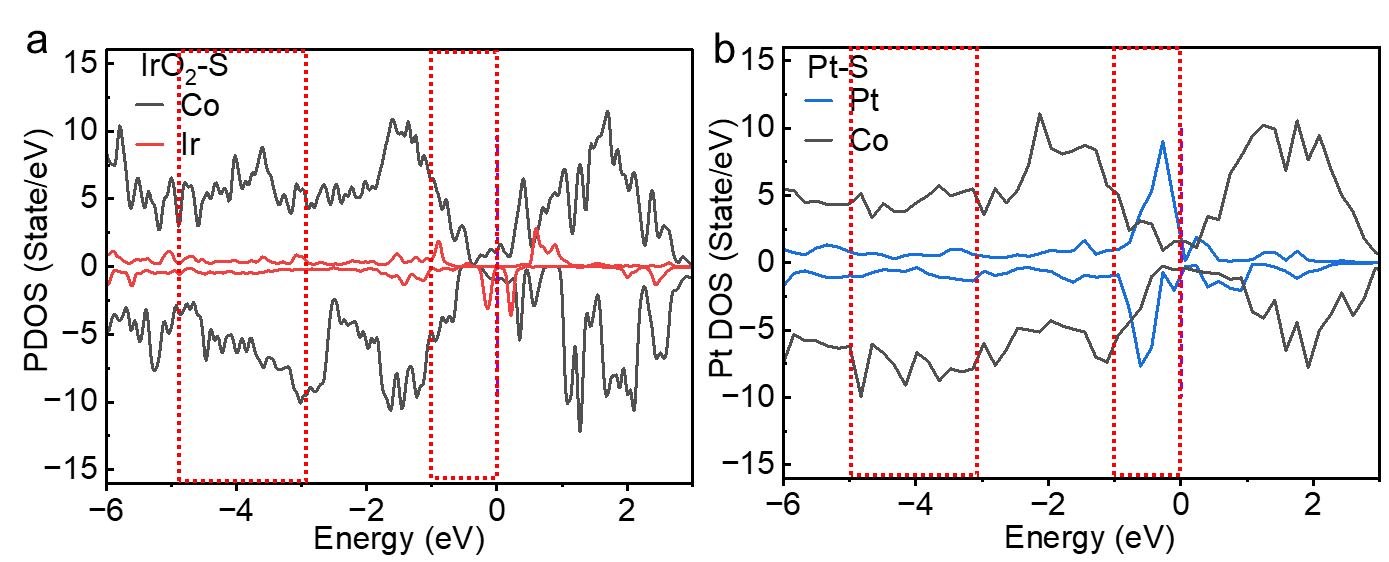


**Fig. S25** The projected density of state (PDOS) of **a** IrO_2_-S and **b** Pt-S


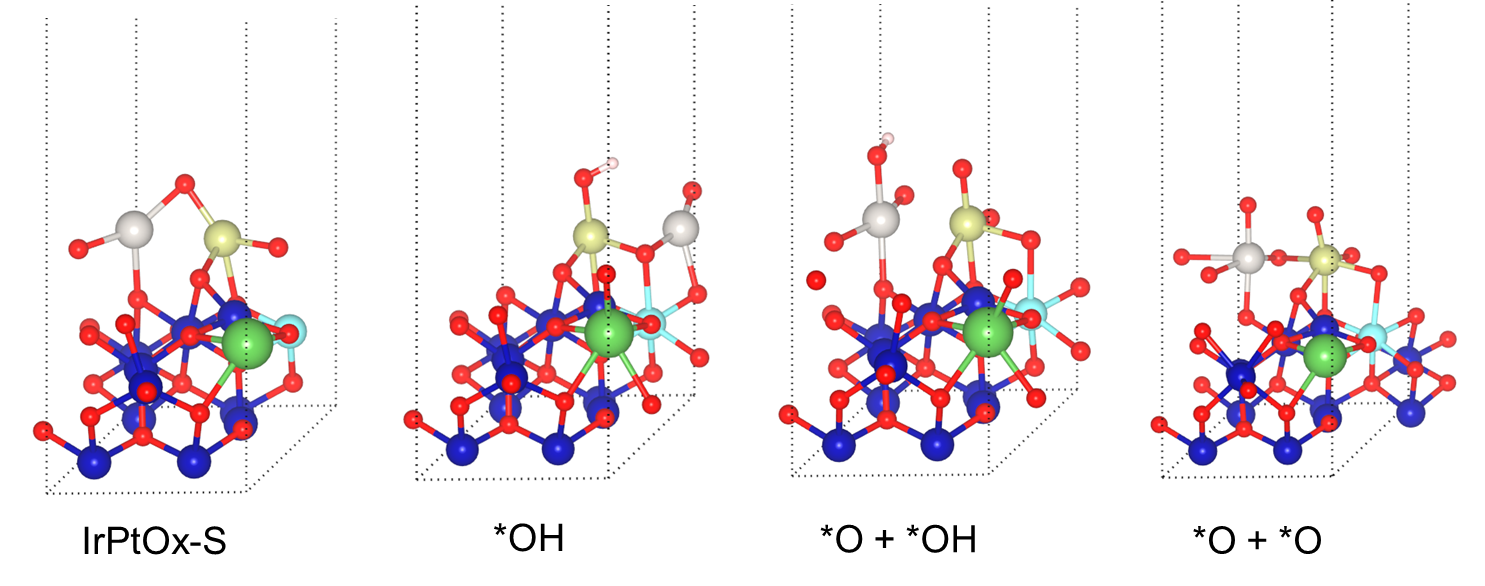


**Fig. S26** OER mechanisms on Ir-O-Pt site of IrPtOx-S that go through *OH, *O+*OH, *O+*O, respectively. Dark blue, red, green, light blue, grey and light-yellow balls represent Co, O, La, Ni, Pt and Ir, respectively


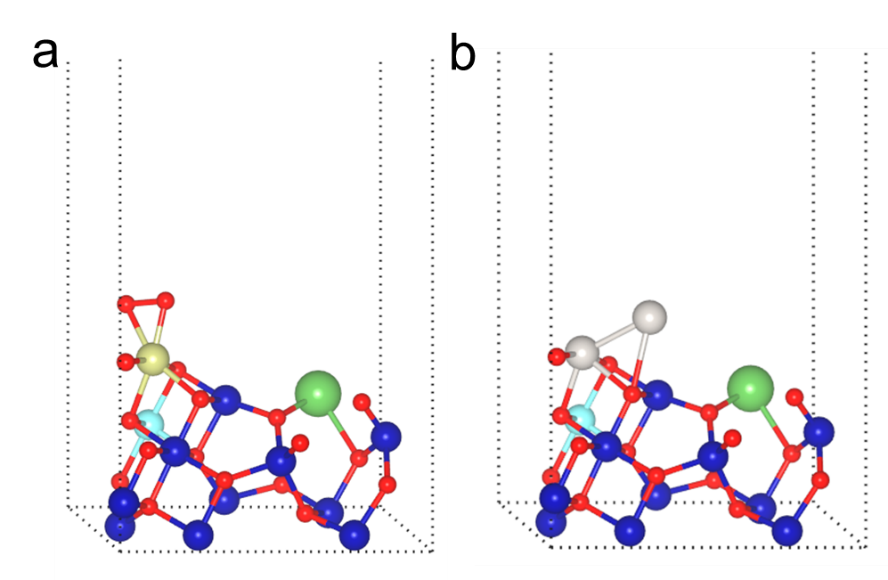


**Fig. S27** The structure of IrO_2_-S and Pt-S used for the simulation. Dark blue, red, green, light blue, grey and light-yellow balls represent Co, O, La, Ni, Pt and Ir, respectively


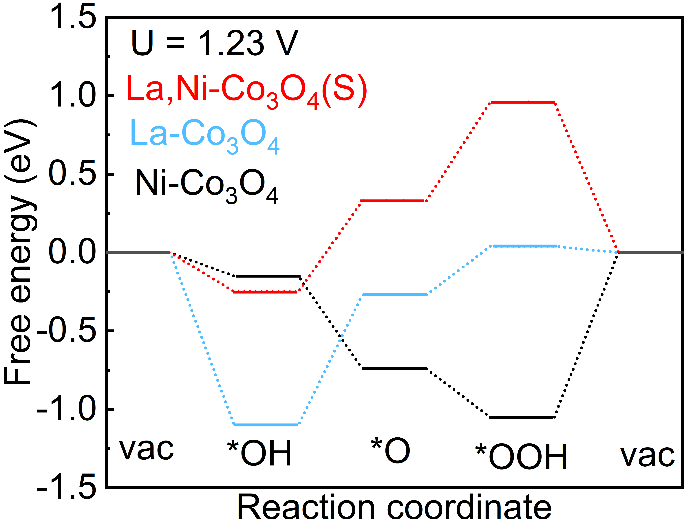


**Fig. S28** Gibbs free energy diagrams of OER on different samples. The calculated energy profiles indicate overpotentials of 0.63 V, 0.83 V, and 1.05 V for La,Ni-Co₃O₄, La-Co₃O₄, and Ni-Co₃O₄, respectively, demonstrating the intrinsic oxygen evolution reaction (OER) activity of these catalysts. These computational results align with our experimental observations, which confirm the activity trend: Ni-Co₃O₄ < La-Co₃O₄ < La,Ni-Co₃O₄

Beside functioning as protective layer (La) and the electronic optimizer (Ni), both La and Ni could optimize the local atomic structure of Co, thus improve the catalytic activity of the LaNi-Co_3_O_4_. The high OER/HER activity of IrPtOx-S, therefore, may stem from the synergy between IrPtOx and the LaNi-Co_3_O_4_ substrate.

**Table S1** Elemental analysis of the material composition measured by ICP-MS

| Elements | Weight concentration |
| --- | --- |
| Co | 31.884% |
| La | 0.818% |
| Ni | 0.2% |
| Ir | 6.738% |
| Pt | 6.779% |

**Table S2** BET surface area, average pore size and volume data from N_2_ adsorption isotherms experiment

| Sample | BET (m^2^ g^-1^) | Main pore diameter (nm) | Pore volume (cm^3^ g^-1^) |
| --- | --- | --- | --- |
| Support (S) | 186.0 | 7.6 | 0.38 |
| IrPtOx-S | 144.5 | 5.5 | 0.30 |

**Table S3** Comparison of overpotential at 10 mA cm^-2^ current density, mass activity (MA) at 300 mV overpotential, durability at constant 10 mA cm^-2^ for OER; overpotential at -10 mA cm^-2^ current density, mass activity (MA) at 50 mV and 100 mV overpotential, durability at constant -10 mA cm^-2^ for HER, as well as the comparison of PEMWE cell performance including activity and durability of IrPtOx-S to that of previously reported Ir- and Pt- based catalysts

| Sample | PGM loading (mg_Ir/Pt_ cm^-2^) | Electrolyte | OER:  η(mV)@10mAcm^-2^ | OER:  MA (A g_Ir_^-1^) @ η=300mV | OER Durability (h) @10mAcm^-2^ | HER:  η (mV) @ -10mAcm^-2^ | HER:  MA (A g_Pt_^-1^) | HER Durability (h) | Cell voltage (V) @loading (mg_Ir_ cm^-2^) @2 Acm^-2^ | MEA Durability | Reference |
| --- | --- | --- | --- | --- | --- | --- | --- | --- | --- | --- | --- |
| IrPtO_x_-S | 0.010 | 0.1M HClO_4_ for OER, 0.5M H_2_SO_4_ for HER | 285.7 | 1344 | 1020 | 57.7±2.5 | 9276 @η=100 mV | 1040@-10 mA cm^-2^ | 1.718@0.075 | 644h@1.8A cm^-2^ | This work |
| Ir_25_Pt_74_ | ~0.07 | 0.5M H_2_SO_4_ for OER and HER | 240 | 1240 | - | 65 | - | 14@-10 mA cm^-2^ | - | - | [S1] |
| ZnNiCoIrMn | 0.28 | 0.1M HClO_4_ for OER and HER | 237 | 610.8 | 100 | 50mV  **@-**50mA cm^-2^ | 675.3 A g_Ir_^-1^ @η=50 mV | 100@-10 mA cm^-2^ | - | - | [S2] |
| A-Ir NS | 0.408 | 0.1M HClO_4_ for OER | 255 | 221.8 | 8 | - | - | - | - | - | [S3] |
| IrO_x_-Ir | 0.133 | 0.5 M H_2_SO_4_ for OER | 293 | ~100 | - | - | - | - | 1.7@1.0(@1.4 A cm^-2^) | 100h@2A cm^-2^ | [S4] |
| HEA@Ir-MEO | 0.15 | 0.05 M H_2_SO_4_ for OER | 243 | 261@350mV |  | - | - | - | 1.7 V@0.4 | 500h@1 A cm^-2^ | [S5] |
| Pt SASs/AG | - | 0.5M H_2_SO_4_ for HER | - | - | - | 12 | 22400 @η=50 mV | 24@-10m Acm^-2^ | - | - | [S6] |
| PtCu/WO_3_@CF | 0.0043 | 0.5M H_2_SO_4_ for HER | - | - | - | 41 | 10860 @η=100 mV | >20@-20m Acm^-2^ | - | - | [S7] |
| Pt/MXene | - | 0.5M H_2_SO_4_ for HER | - | - | - | 34 | 1847 @η=50 mV | 2.7 | - | - | [S8] |
| Pt-N_3_S_1_ SAs |  | 0.5M H_2_SO_4_ for HER | - | - | - | 26 | 13716 @η=50 mV | 50@-10m Acm^-2^ | - | - | [S9] |
| Ir^Ⅵ^-ado | 0.02 | 0.5M H_2_SO_4_ for OER | - | 1150±2 | - | - | - | - | 1.76@0.08 | 2800h@1.8 A cm^-2^ | [S10] |
| IrCoOx@LLCF | 0.010 | 0.1M HClO_4_ for OER | 280 | 1167 ± 150 | 233 | - | - | - | 1.75@0.2 | 246h@2 A cm^-2^ | [S11] |
| Ir-3MA | 0.15 | - | 243 | 261 | 24@100 mAcm^-2^ | - | - | - | 1.79@0.5 | 400h@2 A cm^-2^ | [S12] |
| Ir_0.6_Sn_0.4_O_2_ | - | - | - | - | - | - | - | - | 1.96@0.294 | 100h@1 A cm^-2^ | [S13] |

**Table S4** ECSA of different samples obtained by using H-UPD and Redox-Active Surface Charge methods, respectively

| Sample | ECSA (m^2^ g^-1^) | Method |
| --- | --- | --- |
| IrPtOx-S | 123.1 | H-UPD |
| Ir-S | 45.6 | Redox-Active Surface Charg |
| Pt-S | 96.6 | H-UPD |
| IrO_2_ | 15.1 | Redox-Active Surface Charge |
| Pt/C | 89.6 | H-UPD |

**Table S5** Ir L_III_ edge EXAFS fitting results of IrPtOx-S and IrO_2_. k = 3 – 11 Å^-1^

| Sample | bond | N | R (Å) | σ^2^ (Å^2^) | S_0_^2^ | R-factor |
| --- | --- | --- | --- | --- | --- | --- |
| IrPtOx-S | Ir-Ir/Pt | 11.5 | 2.75 | 0.014 | 0.898 | 0.06 |
| IrO_2_ | Ir-Ir | 2.3 | 3.22 | 0.007 | 0.898 | 0.09 |
| Ir foil | Ir-Ir | 12 |  |  |  |  |
| IrO_2_ | Ir-O | 6 |  |  |  |  |
|  | Ir-Ir_1_ | 2 |  |  |  |  |

**Table S6** Bader charge of Pt/Ir in different chemical environments of slab

| Bader charge | Pt | Ir |
| --- | --- | --- |
| Pt-O-Pt | 0.73 | - |
| Ir-O-Ir | - | 1.17 |
| Pt-O-Ir | 0.71 | 1.54 |

**Supplementary References**

1. C. Liu, I. Roh, H.S. Park, B.J. Park, T. Yu, IrPt alloy nanoparticles with controllable compositions as catalysts for electrochemical oxygen and hydrogen evolution. ACS Appl. Nano Mater. **5**(11), 17152–17158 (2022). <https://doi.org/10.1021/acsanm.2c04069>
2. J. Kwon, S. Sun, S. Choi, K. Lee, S. Jo et al., Tailored electronic structure of Ir in high entropy alloy for highly active and durable bifunctional electrocatalyst for water splitting under an acidic environment. Adv. Mater. **35**(26), 2300091 (2023). <https://doi.org/10.1002/adma.202300091>
3. G. Wu, X. Zheng, P. Cui, H. Jiang, X. Wang et al., A general synthesis approach for amorphous noble metal nanosheets. Nat. Commun. **10**(1), 4855 (2019). <https://doi.org/10.1038/s41467-019-12859-2>
4. P. Lettenmeier, L. Wang, U. Golla-Schindler, P. Gazdzicki, N.A. Cañas et al., Nanosized IrOx–Ir catalyst with relevant activity for anodes of proton exchange membrane electrolysis produced by a cost-effective procedure. Angew. Chem. Int. Ed. **55**(2), 742–746 (2016). <https://doi.org/10.1002/anie.201507626>
5. L. Yao, F. Zhang, S. Yang, H. Zhang, Y. Li et al., Sub-2 nm IrRuNiMoCo high-entropy alloy with iridium-rich medium-entropy oxide shell to boost acidic oxygen evolution. Adv. Mater. **36**(25), 2314049 (2024). <https://doi.org/10.1002/adma.202314049>
6. S. Ye, F. Luo, Q. Zhang, P. Zhang, T. Xu et al., Highly stable single Pt atomic sites anchored on aniline-stacked graphene for hydrogen evolution reaction. Energy Environ. Sci. **12**(3), 1000–1007 (2019). <https://doi.org/10.1039/C8EE02888E>
7. L. Liu, Y. Wang, Y. Zhao, Y. Wang, Z. Zhang et al., Ultrahigh Pt-mass-activity hydrogen evolution catalyst electrodeposited from bulk Pt. Adv. Funct. Mater. **32**(20), 2112207 (2022). <https://doi.org/10.1002/adfm.202112207>
8. Y. Wu, W. Wei, R. Yu, L. Xia, X. Hong et al., Anchoring sub-nanometer Pt clusters on crumpled paper-like MXene enables high hydrogen evolution mass activity. Adv. Funct. Mater. **32**(17), 2110910 (2022). <https://doi.org/10.1002/adfm.202110910>
9. M. Wang, C. Feng, W. Mi, M. Guo, Z. Guan et al., Defect-induced electron redistribution between Pt-N3S1 single atomic sites and Pt clusters for synergistic electrocatalytic hydrogen production with ultra-high mass activity. Adv. Funct. Mater. **34**(2), 2309474 (2024). <https://doi.org/10.1002/adfm.202309474>
10. A. Li, S. Kong, K. Adachi, H. Ooka, K. Fushimi et al., Atomically dispersed hexavalent iridium oxide from MnO_2_ reduction for oxygen evolution catalysis. Science **384**(6696), 666–670 (2024). <https://doi.org/10.1126/science.adg5193>
11. L. Chong, J. Wen, E. Song, Z. Yang, I.D. Bloom et al., Synergistic Co─Ir/Ru composite electrocatalysts impart efficient and durable oxygen evolution catalysis in acid. Adv. Energy Mater. **13**(37), 2302306 (2023). https://doi.org/10.1002/aenm.202302306
12. J. Liang, C. Fu, S. Hwang, C. Dun, L. Luo et al., Constructing highly porous low iridium anode catalysts *via* dealloying for proton exchange membrane water electrolyzers. Adv. Mater. **37**(4), 2409386 (2025). <https://doi.org/10.1002/adma.202409386>
13. G. Jiang, H. Yu, J. Hao, J. Chi, Z. Fan et al., An effective oxygen electrode based on Ir_0.6_Sn_0.4_O_2_ for PEM water electrolyzers. J. Energy Chem. **39**, 23–28 (2019). <https://doi.org/10.1016/j.jechem.2019.01.011>
